# Integrative transcriptome and genome resequencing reveals conserved flowering regulators and allelic variants in early- and late-flowering linseed (*Linum usitatissimum* L.) accessions

**DOI:** 10.1038/s41598-026-40729-7

**Published:** 2026-03-02

**Authors:** Deepa Pal, Daniya Shahid, Ankit Saroha, Akash, Vikender Kaur, J. Aravind, M. Z. Abdin, S. Rajkumar, Rakesh Singh, Gyanendra Pratap Singh, Dhammaprakash Pandhari Wankhede

**Affiliations:** 1 ICAR-National Bureau of Plant Genetic Resources, New Delhi, India; 2 Department of Biotecnology, School of Chemical and Life Sciences, Jamia Hamdard, New Delhi, India

**Keywords:** Flowering, Early flowering, Transcriptome, Candidate genes, Flowering genes, Alleles, Biotechnology, Genetics, Molecular biology, Plant sciences

## Abstract

Flaxseed/Linseed (*Linum usitatissimum*) is an annual crop of economic importance due to its diverse applications in food, nutraceuticals, and industry. Flowering time is a crucial trait in linseed; however, the gene expression landscapes underlying its regulation remain poorly characterized, constraining its understanding and effective application in crop improvement. Here, an integrative transcriptome and genome resequencing approach was used to identify key flowering regulators and allelic variants. Transcriptome sequencing of reproductive tissues including floral bud at two developmental stages, flower, and two vegetative tissues (leaf and stem) in two early flowering linseed genotypes, IC0523807 and IC0525939 were performed. A total of 47.4 GB of filtered data was obtained from 20 datasets, resulting in 34,869 mapped transcripts. Differential expression analysis between vegetative tissues vs. reproductive tissues in different combinations revealed a total of 14,244 differentially expressed genes, with 67 GO and 161 KEGG enriched terms. Several DEGs were involved in auxin, cytokinin, gibberellic acid, and abscisic acid signal transduction pathways. From the 109 major *Arabidopsis* flowering gene orthologs identified in linseed, 54 were differentially expressed, whereas 46 of the 143 putative candidate genes reported in earlier association studies were also represented among the differentially expressed genes. Overall, by integrating a three-way strategy involving differential gene expression, flowering gene orthologs, and candidate genes for flowering, three promising genes were identified, namely, *flowering locus T (FT)*, the flowering repressor *APETALA2-like transcription factor SCHLAFMÜTZE* (*SMZ*), and a *Dof-type domain-containing protein (CDF3)*. The whole-genome resequencing of two early- and late-flowering genotypes was performed, unravelling allelic variations in key flowering candidate genes, including *AGAMOUS-like 19 (AGL19)*,* della protein*,* flowering locus K (FLK)*,* Target of EAT1 (TOE1)*, and *Late Elongated Hypocotyl (LHY)*. This study provides a detailed insight into gene expression profile of early-flowering linseed genotypes, highlighting key flowering pathways, regulators and allelic variants of flowering associated genes.

**Supplementary Information:**

The online version contains supplementary material available at 10.1038/s41598-026-40729-7.

## Introduction

Flowering time is a crucial determinant of reproductive success and seed yield in crop plants. It has been one of the target traits for selection to tailor crop cultivars suitable for a range of eco-geographical areas and to harness the maximum possible yield advantage^1^. Often, flowering under the favourable environments renders advantage of higher seed yield owing to the higher accumulation and partitioning of resources in the extended vegetative growth phase. Early flowering on other hand is preferred in stressful, uncertain environments and short growing seasons^2^.

Linseed/flaxseed (*Linum usitatissimum* L.) is a commercially important oilseed crop in several countries across the globe. It is also known for the diverse applications in food, nutraceuticals, and paint industries^3,4^. It is a facultative long day plant which flowers earlier in long days, than under short days^5,6^. Early flowering is a desirable trait in linseed in several areas of linseed growing countries^7–9^. In Indian conditions, especially in central India with limited water resources, early flowering helps avoid terminal heat, and drought. It is also suitable for linseed cultivation under rainfed conditions and in rice fallows supporting the sustainable utilization of resources^10^. In the other leading linseed growing countries such as Canada, early flowering, maturity, and photo-period insensitivity traits have been considered crucial to expand the growing range of flax to the northern prairie^11^. Further, the changing climatic conditions pose serious challenge for sustainable crop production^12^. Consequently, it is vital to have varieties with a range of flowering and maturity time to ensure maximum yield for different niche areas, latitudes and cropping seasons^2,13^. Flowering is a complex trait and controlled by environmental cues and developmental regulation through an intricate network of signalling pathways^2,5,14^. The genetic pathways underlying floral initiation in the model plant *Arabidopsis* have been substantially unravelled^14–16^. Vernalization, photoperiod & circadian pathway, autonomous, gibberellin (GA), ambient temperature, and age-dependent pathways are among the six flowering pathways identified in *Arabidopsis* that rely on the up- and down-regulation of certain important genes like *FT*,* SUPPRESSOR OF OVEREXPRESSION OF CONSTANS 1 (SOC1)*,* LEAFY (LFY)*, and *APETALA1*^17–19^. In photoperiod pathway, flowering is promoted in the long days, while there is little effect in short days and the pathway involves crucial genes such as *CRYPTOCHROME (CRY2)*,* FLOWERING LOCUS D (FD)*,* FT*,* CONSTANS (CO)*,* GIGANTEA (GI)*,* FE*, and *FWA*^20,21^. Mutation in the genes of the autonomous pathway such as *CA*,* FPA*,* FVE*,* FY*, and *LUMINIDEPENDENS (LD)* show delay in flowering in both long and short days^20^.

Several molecules and pathways are crucial to the process of floral organogenesis. For instance, it is known that sucrose, an important sugar molecule, mediates flowering in a number of plant species^22^. Total starch and sucrose levels in leaf exudate are increased in *Arabidopsis* when flowering is induced by long day (LD) treatment (16 h light/8 h dark)^23^. While sucrose is more strongly linked to maturation, flowering and development of storage organs, glucose primarily affects seedling growth, photosynthesis, starch degradation, and senescence^24^. Similarly, several theories explain how hormones influence flowering^25,26^, flowering gene expression^27^, and flower bud differentiation^28^. Auxin is also essential for the growth of flower primordia^29^. On the other hand, GA controls a number of pathways that impact photoperiodism, stress reactions, flower induction, and autonomous pathways^30^.

In linseed, flowering initiation is known to be influenced by both photoperiod as well as temperature and a considerable genotypic variation in photoperiod sensitivity exists among germplasm accessions^6,9,31,32^. However, there have been limited reports on genetic dissection of flowering time trait in linseed^13,33,36^. Recent association studies have identified 27 QTLs and 53 QTNs related to flowering time in linseed, utilizing accessions from the Canadian core collection and the National Genebank (NGB), India^13,36^. Potential candidate genes identified from the GWAS studies include *FT*,* FD*, the transcriptional regulator *SUPERMAN*,* gibberellin 2-beta-dioxygenase 2*,* HUA ENHANCER 1*,* POLLENLESS 3*,* the photo-responsive gene F-box of flowering 2*,* F-box protein*, and *E3 ubiquitin-protein ligase APD2*^13,36^. These candidate genes have not yet been investigated in detail using integrated approaches, and only limited transcriptomic studies have been conducted to understand the flowering time trait in linseed. Recently, transcriptome sequencing of the flax cultivar ‘Royal’ identified differential expression of known *Arabidopsis* flowering genes and highlighted major regulators, including *SOC1*,* FUL*,* AP1*, members of the *MADS-box (MIKC class)*, and *SBP* transcription factor families^11^. However, transcriptome profiles of tissues such as leaves and stem, which are crucial for perceiving external and internal cues (e.g., inductive photoperiod, hormones), generating the flowering signal, and transporting it *via* phloem to shoot apical meristem for floral transition have not been explored in linseed so far.

As, early flowering is an important breeding objective in linseed, achieving this requires identification of key candidate genes underlying flowering-time variation and characterization of their allelic diversity, which remain largely unexplored at present. Accordingly, the present study is aimed to study transcriptomic profiles of two early flowering genotypes using vegetative and reproductive tissues to capture floral signal generation in leaves and its expression in developing buds and flowers. Furthermore, the differentially expressed genes identified in this study were integrated with candidate genes from previous association studies on flowering time in linseed, along with orthologs of flowering genes from *Arabidopsis thaliana*, to identify key candidate genes. Consequently, allelic variation in selected flowering-related genes was assessed between early- and late-flowering genotypes. This integrated approach offers valuable insights into the transcription network associated with flowering time regulation in linseed.

## Materials and methods

### Plant material, RNA isolation, cDNA library preparation and sequencing

**Fig. 1:**
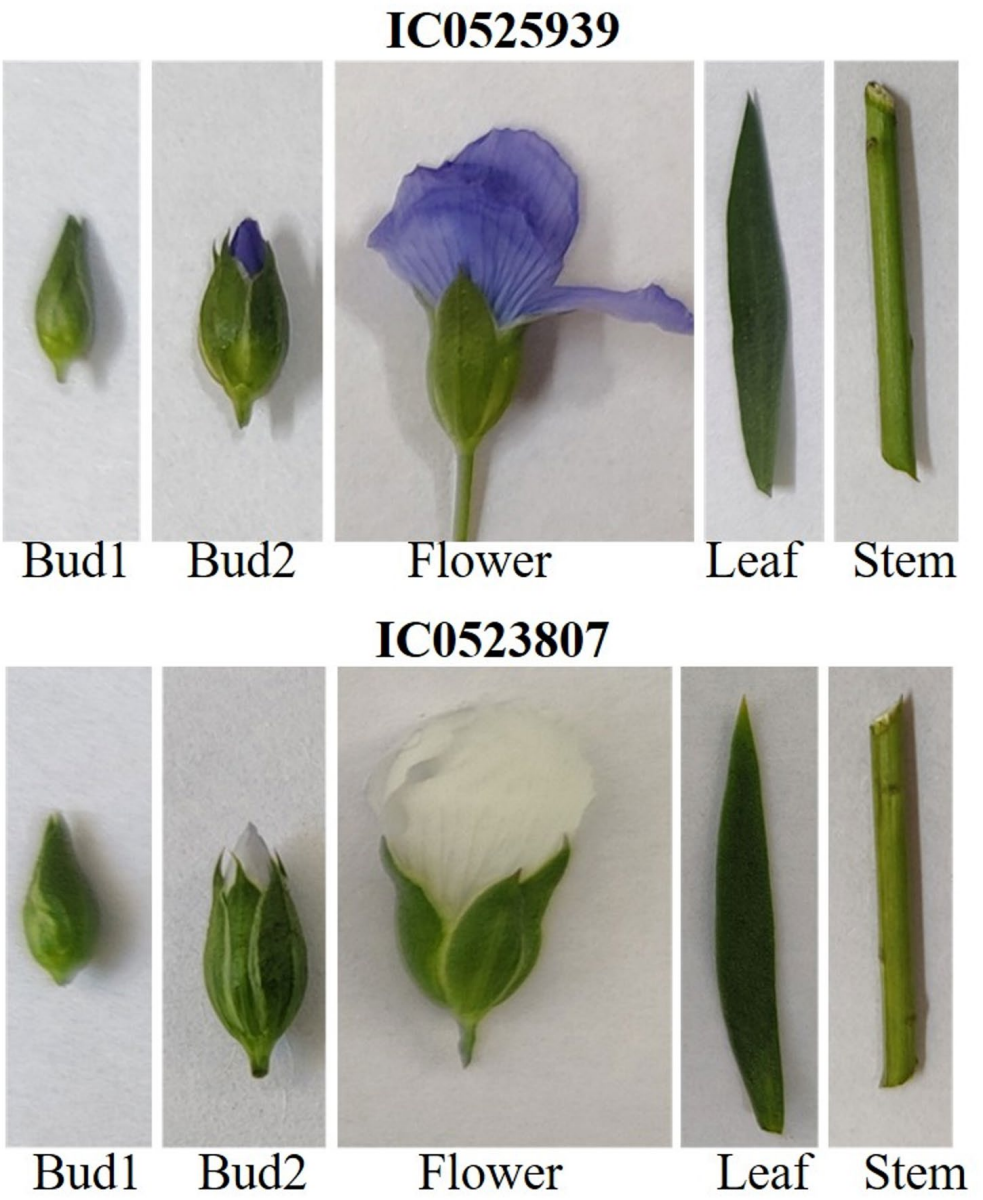
Floral and vegetative tissues of early flowering accessions (IC0525939 and IC0523807) used for transcriptomics studies (Images are representative and shown without scale).

Two early flowering genotypes from NGB accession numbers namely, IC0523807 and IC0525939 were selected for transcriptome study based on earlier phenotypic multi-location evaluation^32,36^. The seeds of the samples were obtained from NGB, Indian Council of Agricultural Research-National Bureau of Plant Genetic Resources, New Delhi, India. IC0525939 is a landrace originally collected from Lalitpur, Uttar Pradesh, India in 2005, whereas, IC0523807 is released variety *Sharda* (LMS-4-27). The seed samples of both the genotypes have been conserved in NGB with above mentioned national identity numbers. The seed samples from NGB, India are accessible for research purpose following the standard material transfer agreement and as per the terms, conditions (https://nbpgr.org.in/nbpgr2023/germplasm-exchange-2/). Samples of the selected early flowering genotypes were collected from the field experiment laid out in an augmented block design (ABD) at NBPGR research farm, Indian Agricultural Research Institute, New Delhi, India (28.64°N, 77.15°E)^36^. Seeds derived from single-plant progeny of both genotypes were used for sowing. Samples of floral buds at two developmental stages, bud1- stage 5 (petal growth accelerated, styles begin to grow upward.), bud2- stage 8 (petals enclose inner whorls, flask-shaped), flower- stage12 (anthesis, petals reflexed)^37^, leaves and stem (Fig. 1) were collected during flowering stage from field grown plants in the morning of 15th January 2021 (temperature: minimum 13.6 °C & maximum 30.7 °C; relative humidity %: RH I- 68 and RH II 37; rainfall (mm): 0; dawn: 6:50AM and dusk 6:12PM) (Table S1). Samples were collected in two biological replicates each and snap frozen in liquid nitrogen and kept at -80°C until RNA isolation. Total RNA was extracted using TRIzol method with some minor modifications along with DNase treatment as per manufacturer’s instructions. The qualities and quantities of the isolated RNA samples were checked on 1% denaturing RNA agarose gel (intact 28 S, and 18 S ribosomal RNA bands, no high molecular DNA band or smear) and NanoDrop, ThermoFischer Scientific, Waltham, MA, USA (OD A260/280: 1.89–2.01). The quality of RNA was also checked through Agilent 2100 Bioanlyser, Germany and the QC passed samples were used for library preparation. The enrichment of mRNA was done from the total RNA using Poly-T attached magnetic beads, followed by enzymatic fragmentation. First and second strand cDNA synthesis was performed using SuperScript II reverse transcriptase with Act-D mix, and DNA polymerase with second strand mix, respectively. The purification of double-stranded cDNA was achieved using AMPure XP beads. This was followed by A-tailing, adapter ligation, PCR-mediated enrichment, and purification of the enriched libraries with AMPure XP beads. The purified libraries were then analyzed using the 4200 TapeStation system (Agilent Technologies, Santa Clara, CA, USA). A total 20 Ribonucleic acid paired-end sequencing (RNA-Seq) libraries of five samples in two biological replicates for two accessions, IC0523807 and IC0525939 were prepared using TruSeqTM stranded mRNA sample prep kit (Illumina Inc., San Diego, CA, USA). Paired-end sequencing was carried out by an Illumina NovaSeq6000 sequencer (Illumina Inc., San Diego, CA, USA.) to generate raw reads.

### Quality control, read alignment, transcript abundance, and expression analysis

The adapter sequences and low-quality reads were trimmed using Trimmomatic tool v0.39^38^. The cleaned reads were aligned to the linseed reference genome (https://phytozome.jgi.doe.g.ov/pz/portal.html#!info?alias=Org_Lusitatissimum) using Hierarchical Indexing for Spliced Alignment of Transcripts (HISAT2) tool v2.2.1 using default parameters^39,40^. The aligned reads were used for transcriptome assembly using StringTie tool v2.2.3^40,41^. Transcript abundances in the form of TPM (Transcripts per Million) were quantified using Salmon tool v1.10.1^42^, a tool for transcript-level quantification. The ‘tximport’ R package v1.31.1^43^ was used for downstream analysis and integration of transcript abundance data. Differential expression analysis between specific conditions was performed using DESeq2 tool v1.45.1^44^. Genes were identified as differentially expressed based on a p-value and adjusted p-value ≤ 0.05 and a log2 fold change ≥ 2 or ≤ − 2. Pairwise comparisons included bud 1 vs. leaf, bud 2 vs. leaf, flower vs. leaf, bud 1 vs. stem, bud 2 vs. stem, and flower vs. stem for both linseed accessions IC0523807 and IC0525939. Volcano plots were generated by plotting the log2 fold change against the −log10(adjusted p-value) to visualize differentially expressed genes (DEGs) among comparisons using a custom script in ‘R’.

### Annotation, GO, and KEGG analysis

For annotations of all transcripts, BLASTx alignment was performed with an e-value threshold of 1.0e-05 against protein databases including NR (NCBI non-redundant protein), InterPro, and KEGG. GO annotation of the transcripts was performed by Blast2GO software^45^ to classify the transcripts based on molecular functions, biological processes, and cellular components.

To understand the role of DEGs in different biological processes, GO and KEGG enrichment analyses were performed using the ‘enricher’ function of the clusterProfiler v4.12.0 package of R^46,47^. A significance threshold of *p* < 0.05 was applied to determine the statistical significance of enriched GO terms and KEGG pathways. Enriched GO terms were categorized into biological processes, cellular components, and molecular functions. This facilitated the identification of overrepresented terms within our list of DEGs for clear interpretation of observed transcriptional changes in DEGs. The identified enriched KEGG terms were used as input to conduct detailed pathway analysis using KEGG mapper (https://www.genome.jp/kegg/mapper/reconstruct.html) to further investigate what genes are expressed in some specific pathways. MapMan annotation file was created using Mercator web application^48^ and was used as a mapping file for data visualization in MapMan software v3.5.1R2^49^.

### Expression of known flowering genes and putative candidate genes from GWAS studies

The gene sequences of known flowering genes in *Arabidopsis* were used to identify the respective orthologs in linseed through a BLAST search (best hit) against the total reported genes in the linseed reference genome. Simultaneously, the reported putative candidate genes for flowering time traits in linseed were selected from the genome-wide association studies (GWAS) from two recent reports^13,36^. Gene expression of the selected genes were studied across different tissue types in the present study and considered differentially expressed if the expression exceeded the threshold (p-value < 0.05 and log2-fold change ≥ 2 or ≤ -2). The three complementary approaches, DEG analysis, GWAS-based candidate gene information, and orthology with known *Arabidopsis* flowering genes, were integrated to identify common candidates which were considered as key genes for flowering regulation in linseed.

### Whole genome resequencing and allele mining

To study the allelic variations in key genes, two early-flowering (IC0523807, IC0525939) and two late-flowering accessions (EC0115148, EC0718827) were selected for whole-genome resequencing. Seeds of the above-mentioned accessions were obtained from NGB, India. Seeds from single-plant progeny of all four accessions were used for plant raising and DNA isolation. EC0115148 and EC0718827 were originally procured from the United States and Australia, respectively, through NGB, India. DNA isolation was performed using the Qiagen DNeasy Plant Mini kit. DNA quality was checked on a 1% agarose gel, and concentration was estimated using the Qubit DNA HS kit (Thermo Scientific, Cat. No: Q33230). DNA libraries were prepared using the QIAseq FX DNA Library Kit (Cat. No: 180479) according to the manufacturer’s instructions. Library quality control was done using Agilent D1000 screentape (Agilent, Cat No. 5067–5582) and Qubit™ 1X dsDNA High Sensitivity (HS) (Thermo, Q33230). The libraries were sequenced on the Illumina NovaSeq platform.

The quality of sequencing reads was assessed using the FastQC tool v0.12.1^50^. Low-quality reads, adapter content, and other contaminants were removed using Trimmomatic tool v0.39^38^. Cleaned reads were aligned to the reference genome (ASM22429v2) (www.ncbi.nlm.nih.gov/datasets/genome/GCA_000224295.2/) using the ‘very-sensitive-local’ algorithm of Bowtie2 tool v2.5.4^51^. The resulting alignment files, in SAM format, were converted to BAM format and subsequently sorted, filtered, and indexed using SAMtools v1.21^52^. SNP variant calling was performed using the mpileup algorithm in BCFtools v1.21^52^. Variants were filtered based on a quality score ≥ 30 and read depth ≥ 5. Finally, the filtered variants were superimposed onto the reference genome using BCFtools, and fasta files were constructed for the respective accessions. The flowering gene sequences were retrieved according to the genomic coordinates of the CDC Bethune reference genome^35,53^. Genes from the reference and linseed accessions were aligned using MAFFT v7.526^54^, and variants were filtered.

### 3-D Homology modelling and analysis of variant protein structure

SWISS-MODEL server was used to obtain the wild type protein structure through homology modelling^55^. For the mutant protein structure and visualization PyMOL (Schrödinger, LLC) was used. The GalaxyRefine tool^56^ was used to refine the modelled structures by minimizing steric clashes and improving the structural quality. Molecular dynamics simulations were performed using GROMACS 2023^57^. The system was solvated in a TIP3P water model within a dodecahedral box. The potential energy and RMSD (Root Mean Square Deviation) values were compared between the wild-type and mutant structures to assess mutation stability.

## Results

### Transcriptome overview and quality metrics

The transcriptome analysis of linseed was conducted in three reproductive tissues (two floral bud developmental stages- bud1, bud2, and flower), and two vegetative tissues (leaf, and stem) in two early flowering linseed accessions IC0523807 (variety- Sharda) and IC0525939 (germplasm) (Fig. 1). A total of 20 mRNA libraries of five tissues in two biological replicates for two accessions were constructed and sequenced. After removing the low quality and contaminant reads, a total of 639.3 million clean reads were obtained, with an average of 31.96 million reads per sample. On average, 95.26% of the input reads mapped uniquely to the *L. usitatissimum* reference genome (CDC Bethune). A summary of the sequencing data is presented in Table 1. The sequencing data of the present study have been deposited in Sequence Read Archive (SRA) database of National Center for Biotechnology Information (NCBI) with the Bio Project ID- PRJNA773597. A threshold of < 1 TPM transcript abundance in all the tissues were considered as not expressed genes and filtered out. Accordingly, a total of 34,869 genes from the total 42,277 genes on reference genome were found expressed in either of the tissue types or accessions. All the 34,869 genes could be annotated with InterproScan, whereas 34,215 gene transcripts were found to have blast hits using NR (non-redundant protein database, NCBI). A total of 28,341 transcripts were associated with GO (Gene Ontology) terms, and 17,658 transcripts could be annotated with KEGG (Kyoto Encyclopedia of Genes and Genomes).

**Table 1:** Details of sequence reads in different tissues of two early flowering accessions used in transcriptome study.

Genotype	Tissue	Replicate	Reads after quality control	GC%	Reads mapped to reference (%)
***IC0523807***	Bud 1	R1	26797304	47	96.27
		R2	26188676	46.5	96.37
	Bud2	R1	23975680	49.5	94.07
		R2	27864282	49.5	93.84
	Flower	R1	19525124	47.5	94.57
		R2	25912018	47	96.99
	Leaf	R1	38024594	46.5	95.53
		R2	39092980	46.5	94.40
	Stem	R1	25804440	49	92.57
		R2	24607258	46	95.27
***IC0525939***	Bud 1	R1	41663314	48.5	94.60
		R2	42755052	46.5	96.59
	Bud2	R1	36138224	46.5	96.91
		R2	48620370	48.5	97.04
	Flower	R1	46467562	47.5	96.84
		R2	42365566	47.5	96.92
	Leaf	R1	28588298	48.5	92.18
		R2	33816376	48	93.71
	Stem	R1	19508232	47.5	96.01
		R2	21586792	48	94.52

### Quantification and differential expression analysis

**Fig. 2:**
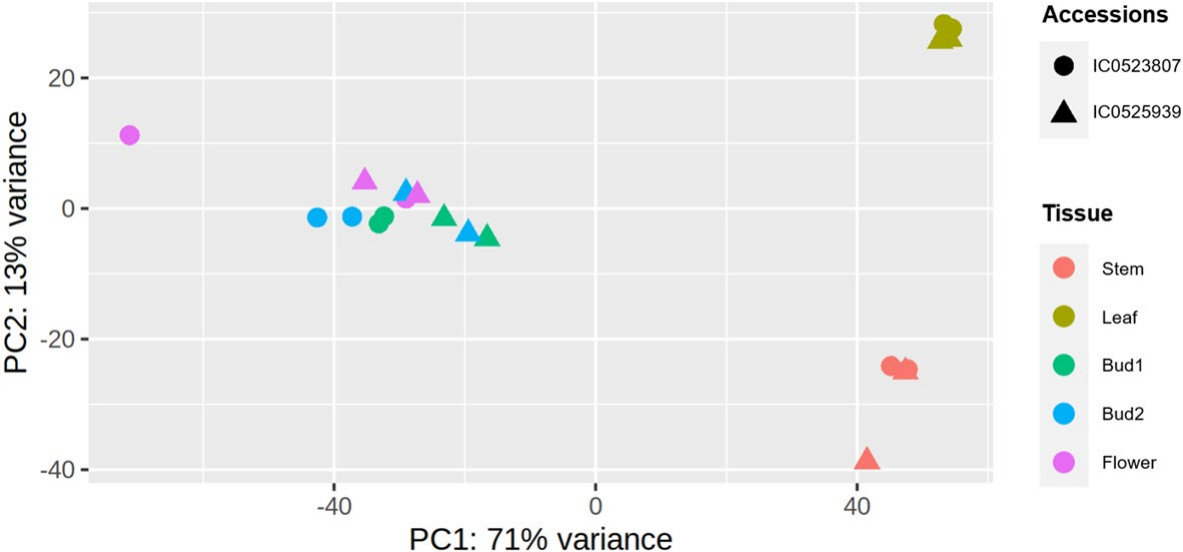
Principal component analysis plot for transcriptome data of biological replicates bud1, bud2, flower, stem, and leaf tissues of early flowering linseed accession IC0525939 and IC0523807.

The TPM (Transcripts per Million) estimate was used to normalize the expression of identified transcripts across different tissues (Table S2) in both the accessions. The consistency of biological replicates was assessed by plotting a PCA based on TPM values of the predicted transcripts (Fig. 2). The PCA plot showed close clustering of replicates from each tissue type of the accession, while different tissue types were positioned further apart, indicating a clear separation between the studied tissue types.

Differential expression analysis was conducted using DESeq2 to identify genes with significant expression differences between reproductive and vegetative tissues in linseed accessions IC0523807 and IC0525939 (Table S3). Comparisons included bud 1 vs. leaf, bud 2 vs. leaf, flower vs. leaf, bud 1 vs. stem, bud 2 vs. stem, and flower vs. stem in both the accessions IC0523807 and IC0525939. The analysis revealed substantial numbers of both upregulated and downregulated genes in each comparison (Fig. S1). In bud1 vs. leaf, 5,541 and 4,955 genes were upregulated, and 2,164 and 2,378 genes were downregulated in IC0523807 and IC0525939, respectively. In bud2 vs. leaf, 5,412 and 5,145 genes were upregulated, and 2,057 and 2,513 were downregulated. In flower vs. leaf, 5,711 and 5,150 genes were upregulated, while 1,829 and 2,380 were downregulated. In bud1 vs. stem, 2,703 and 1,983 genes were up- and downregulated in IC0523807, while in IC0525939, 2,259 and 2,522 genes were up- and downregulated, respectively. The number of up-regulated genes in bud2 vs. stem and flower vs. stem were relatively lower in both accessions, with 2343 and 2703 genes upregulated in IC0523807, while 2130, and 2343 genes upregulated in IC0525939. The number of downregulated genes in bud2 vs. stem and flower vs. stem were 1485 and 1399 in IC0523807, whereas 2195 and 2260 in IC0525939. The high number of DEGs highlights substantial transcript up and downregulation in reproductive and vegetative tissues in both the accessions. The observed small variation in the number of up- and downregulated genes between the two accessions could potentially be influenced by genotype-specific variation reflecting overall phenotypic differences.

Volcano plots were created by plotting the fold change values against the negative log of the p-values to determine the number of transcripts that are significantly differentially expressed in six comparisons (bud1 vs. leaf, bud2 vs. leaf, flower vs. leaf, bud1 vs. stem, bud2 vs. stem, flower vs. stem) in both the early flowering accessions (Fig. S2A-L). Genes common between two accessions for each condition were taken into consideration. The names of the top ten differentially expressed genes have been displayed in each comparison. The list of DEGs and their fold expression in each comparison in two accessions have been given in Table S3. Among the top ten DEGs, 17 (13 up and 4 downregulated) and 15 DEGs (6 up- and 9 downregulated) were observed in at least two of the six comparisons each in accession IC0523807, and IC0525939, respectively. These genes included monooxygenase, putative aquaporin PIP2-8, tubulin, LLDR protein, and several uncharacterized proteins. Many of these genes are generally associated with fundamental cellular processes such as metabolism, water transport, cytoskeletal organization, and stress-related responses, and their differential expression is likely indicative of tissue-specific functional requirements rather than direct involvement in flowering regulation.

**Fig. 3:**
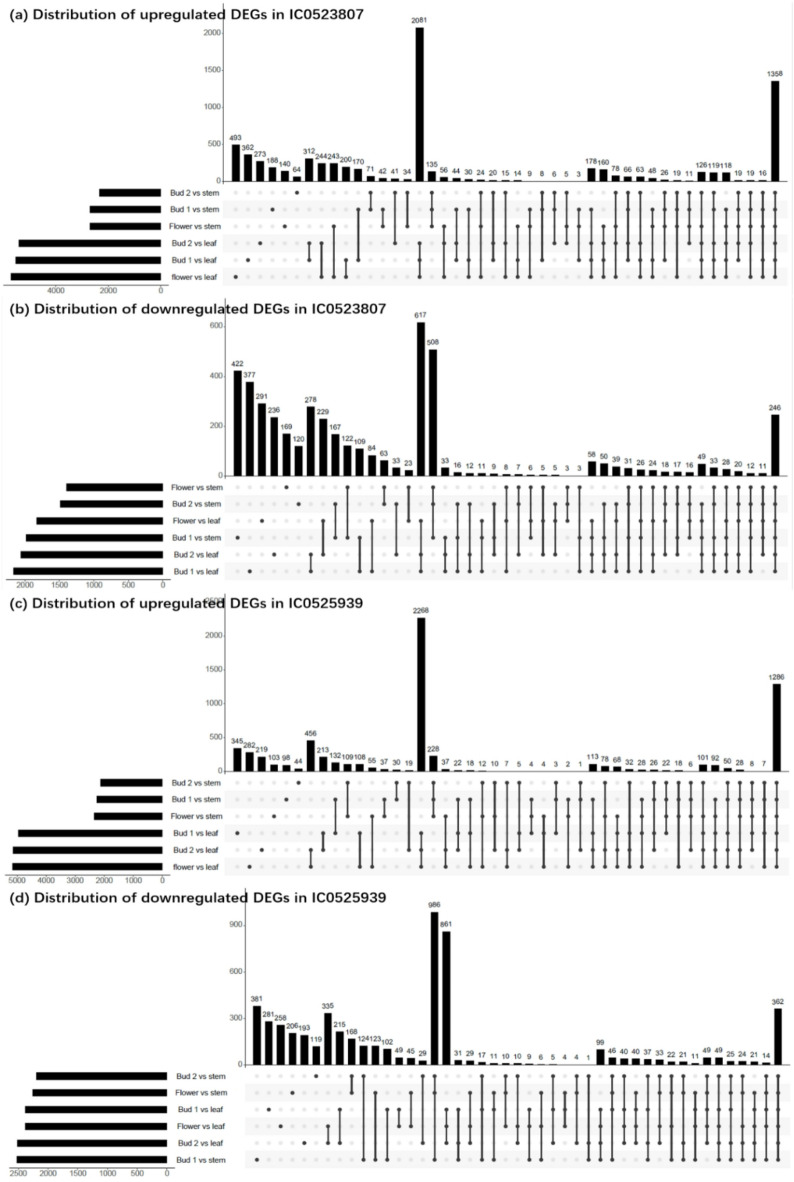
UpSet plot showing the total number of differentially expressed genes (DEGs) across six comparisons between vegetative and reproductive tissues. Up- and down regulated DEGs in early flowering accession IC0523807 (a-b) and IC0525939 (c-d) are shown. The vertical bars indicate the size of intersections of DEGs among the respective comparisons (lines connecting the dots indicate the intersecting conditions). The horizontal bars represent the total number of DEGs for each individual tissue comparison. The DEGs from six tissue-wise comparisons were illustrated across 45 different combinations on the x-axis to specifically depict the upregulated and downregulated genes for each comparison in each accession.

After removing the redundancy among the different conditions, there were 14,244 DEGs between vegetative and reproductive tissues in both the accessions. Among these genes, 7894 were up regulated, while 6350 were down regulated (Table S4). To visualize how many transcripts were common as well as unique among the six specified comparisons, four separate Up-Set plots were created for up regulated and down regulated transcripts for two accessions, IC0523807, and IC0525939 (Fig. 3). Highest number of upregulated DEGs 2081 and 2268 were observed common under ‘bud1 vs. leaf, bud2 vs. leaf, flower vs. leaf’, followed by 1358 and 1286 in all six combinations in accessions IC0523807, and IC0525939, respectively. In contrast, a comparison of reproductive tissues (bud1, bud2, and flower) vs. stem showed substantially less number of common upregulated genes 135 and 228 in IC0523807, and IC0525939, respectively. Whereas, in case of downregulated genes, the highest downregulated genes, 617 were common in reproductive tissues (bud1, bud2, and flower) vs. leaf followed by 508 genes in reproductive tissues (bud1, bud2, and flower) vs. stem in accession IC0523807. In case of IC0525939, a combination of (bud1, bud2, and flower) vs. stem showed highest number of downregulated genes 986 followed by 861 in a combination ‘(bud1, bud2, and flower) vs. leaf’. Accordingly, among the downregulated genes, a combination of (bud1, bud2, and flower) vs. stem showed either the highest and the second highest number of downregulated genes in both accessions. This indicates the comparable up- and downregulation of genes in both the early flowering accessions.

### Global functional landscape of DEGs

To study the significant functions associated with the differentially expressed genes (DEGs), Gene Ontology (GO) and KEGG enrichment analysis was performed. Combined GO enrichment analysis was performed on reproductive tissues (bud 1, bud 2, and flower) vs. leaf and stem tissues in two accessions with a significance threshold set at a p-value  0.05. The analysis revealed total 67 unique enriched GO term, with 40 and 52 enriched GO terms associated with 2,049 and 2,654 DEGs in reproductive tissues vs. leaf and stem tissue, respectively (Table S5). DEGs were represented in three major categories: biological processes, molecular function, and cellular component having 16, 19, and 5 enriched GO terms and 522, 1,281, and 246 associated DEGs, respectively in reproductive tissues vs. leaf. In case of in reproductive tissues vs. stem, there were 12, 31, and 9 enriched GO terms and 493, 1770, and 391 associated DEGs in biological processes, molecular function, and cellular component, respectively. In both comparisons, it was observed that molecular function had the majority of genes (1,281 and 1,770) distributed across functions such as hydrolase activity, followed by monooxygenase and oxidoreductase activity both of which have proven role in flowering regulation.

To investigate which DEGs are activated and suppressed in different pathways, gene expression information was mapped to Kyoto Encyclopedia of Genes and Genomes (KEGG) database (Fig. S3–S5). The DEGs were found to be involved in 161 enriched KEGG pathways (Table S6). The interleukin-1 receptor-associated kinase 4 (K04733) pathway showed highest 158 associated DEGs. The top 20 KEGG enrichment terms included transcription factor *MYB* (K09422), *peroxidase* (K00430), *cytochrome* *P450 family 1 subfamily A1* (K07408), *calcium-binding protein CML* (K13448) and *MADS-box transcription enhancer factor 2 A* (K09260) (Fig. S4). The *MADS-box transcription factors/enhancer* which are known to have role in floral development were found to be in three pathways K09260, K09264, and K04454 with 43, 34 and 15 associated DEGs, respectively. The key multiple pathways which have been identified included *histone H2A* (K11251), *histone H4* (K11254), and *histone H3* (K11253) with 16, 13, and 11 genes, respectively. There were also three enriched pathways of E3 ubiquitin-protein ligase (K19041, K11982, and K22378) with DEGs ranging from 18 to 29. Two auxin-responsive protein related pathways, *auxin-responsive protein IAA* (K14484) and *auxin responsive GH3* gene family (K14487) were identified with 26 and 11 DEGs, respectively. The other important enriched pathways related to flowering time/floral development included *aquaporin PIP* (K09872), *mitogen-activated protein kinase* (K04371), *brassinosteroid 6-oxygenase* (K09590), and *WRKY transcription factor* (K13424) (Table S6).

### Differential regulation of circadian, hormonal, and redox pathways

**Fig. 4:**
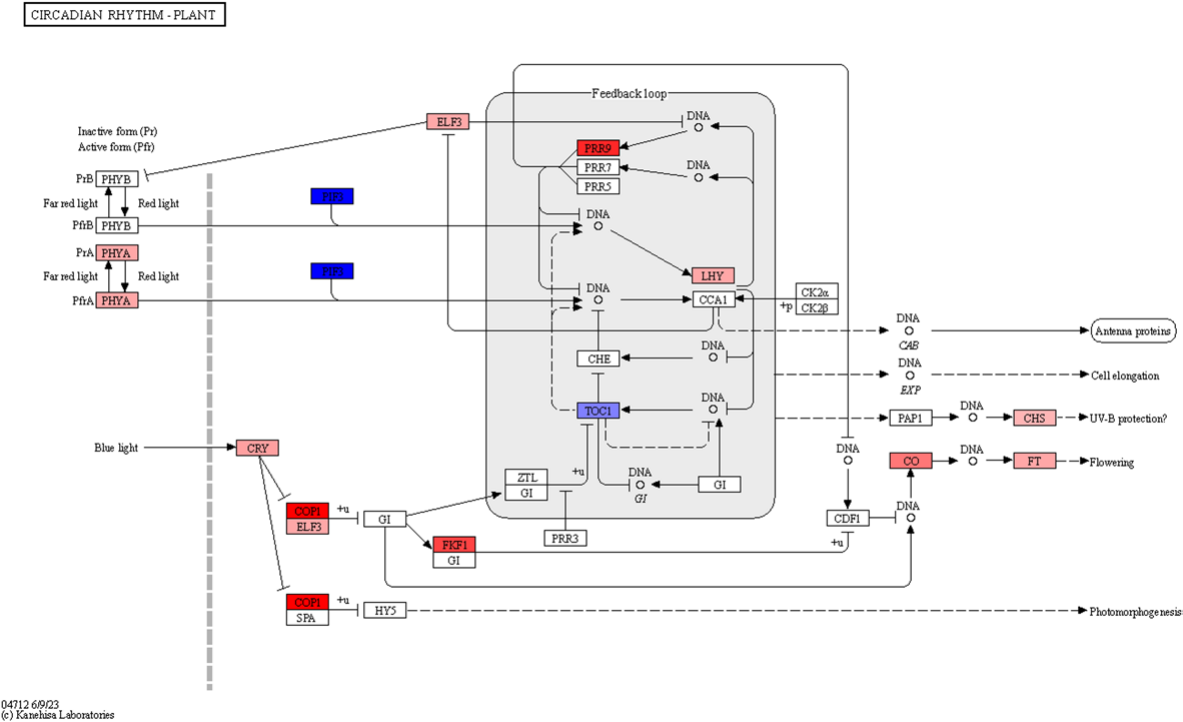
KEGG pathway analyses of differentially expressed genes in plant circadian rhythm pathway. DEGs mapped and displayed in red and blue colour boxes show down- and up-regulation, respectively. White colour box showed the genes were not differentially expressed. PHY-A: Phytochrome A, PHYB: Phytochrome B, PIF3: Phytochrome-Interacting Factor 3, CRY: Cryptochrome (CRY1 or CRY2, blue-light receptor), COP1: Constitutively Photomorphogenic 1, ELF3: Early Flowering 3, GI: GIGANTEA, HY5: ELONGATED HYPOCOTYL 5, FKF1: Flavin-binding, Kelch repeat, F-box 1, CK2α: Casein Kinase 2 alpha subunit, CK2β: Casein Kinase 2 beta subunit, CDF1: Cycling DOF Factor 1, CAB: Chlorophyll A/B-Binding Protein, EXP: Expansin (cell wall-loosening protein), PAP1: Production of Anthocyanin Pigment 1 (MYB transcription factor), CO: CONSTANS, CHS: Chalcone Synthase, FT: FLOWERING LOCUS T, PRR3: Pseudo-Response Regulator 3, PRR5: Pseudo-Response Regulator 5, PPR7: Pentatricopeptide Repeat Protein 7, PPR9: Pentatricopeptide Repeat Protein 9, ZTL: ZEITLUPE (F-box protein), CHE: CCA1 HIKING EXPEDITION, TOC1: TIMING OF CAB EXPRESSION 1, LHY: LATE ELONGATED HYPOCOTYL, CCA1: CIRCADIAN CLOCK ASSOCIATED 1. The up- and downregulated expression profiles of circadian rhythm–related genes in vegetative and reproductive tissues highlight the potential involvement of these DEGs in flowering time regulation through the circadian pathway in the early flowering linseed accessions IC0523807 and IC0525939. ​.

To further examine the DEGs involved in different pathways related to flower development, DEGs were mapped to key flowering regulation pathways including the circadian rhythm pathway, plant hormone signal transduction, and sucrose/starch metabolism using KEGG mapper and MapMan. DEGs mapped on the circadian rhythm pathway (Fig. 4), included *red/far-red light-receptor phytochromes (PHY-A)*,* Early Flowering 3 (ELF3)*,* photoperiodic response rhythm 9 (PRR9)* and *clock gene*,* Late Elongated Hypocotyl (LHY)*. As expected, these genes showed reduced expression in reproductive tissues and higher expression in vegetative tissues, leaf/stem. Similarly, another major photoperiodic photoreceptor, the blue/UV-A light-receptor *cryptochrome (CRY)*,* F-BOX1 protein (FKF1)*,* ELF3*,* constans (CO)*,* ‘florigen’ FT* which promote flowering were also seen in DEGs in circadian pathway and showing lower expression in reproductive tissues over vegetative tissues, leaf/stem. Further, the known repressor of photoperiodic flowering, *Constitutively Photomorphogenic 1 (COP1)*,* a RING-finger E3 ubiquitin ligase* was also among the DEGs in circadian rhythm pathway and also found to have higher expression in vegetative tissues. *PIF3*, which is a known positive regulator *of PHY-A* and *PHY-B* in *Arabidopsis*, and *TOC1 (Timing of Cab Expression1)*, an important component of circadian clock was found to be up regulated in flowering tissues in circadian rhythm pathway. A significant number of DEGs were identified in plant hormone signal transduction including auxin (6 DEGs), cytokinin (5 DEGs), gibberellin (58 DEGs), abscisic acid (236 DEGs), and brassinosteriod (11 DEGs) (Table S7, Fig S5). In addition, significant DEGs were found to be involved in redox homeostasis including ‘heme’, ‘thioredoxin’, ‘ascorb/gluath’, ‘glutaredoxin’, ‘periredoxin’, and ‘dismutase/catalase’ (Fig. S5).

### Gene expression profiles of top DEGs in floral and vegetative tissues

**Fig. 5:**
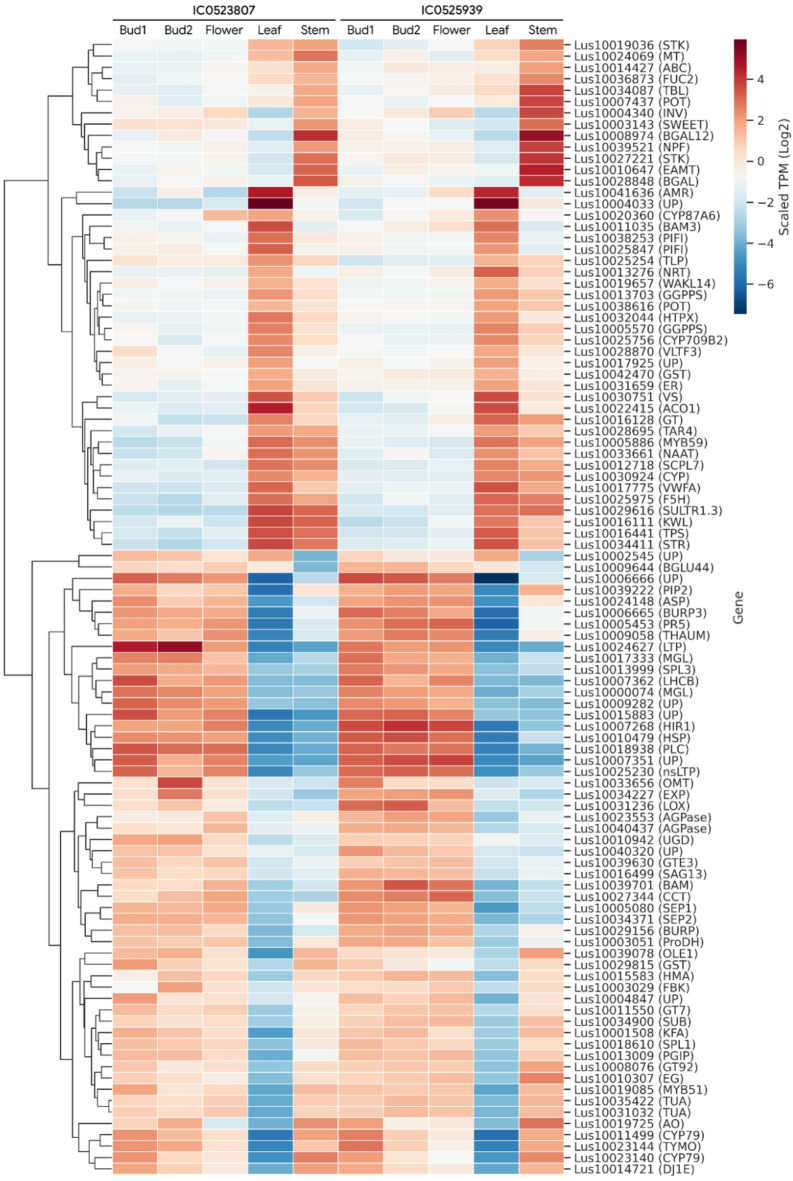
Heat map of gene expression profile of top 100 DEGs in floral and vegetative tissues of early flowering linseed accessions IC0525939 and IC0523807. Locus ids of the genes have been shown on right side and expression-based clustering on left side. Transcript abundances in the form of TPM (Transcripts per Million) in colour gradient is shown.

Gene expression profile of top hundred DEGs were analysed in reproductive and vegetative tissues in both the early flowering accessions (Fig. 5). There were two major clusters of the genes observed based on the expression value, one with higher expression in vegetative tissue (leaf and stem) and the other with higher expression of genes in reproductive tissues (bud1, bud2 and flower). In the first cluster from the two subclusters, 13 genes showed highest expression in stem in both the accessions, which included *NPF family transporter *(*Lus10039521)*,* proton-dependent oligopeptide transporter (Lus10007437)*,* beta-galactosidase 12 (Lus10008974)*,* metal transporter (Lus10024069)*,* ABC transporter B family member 1 (Lus10014427)*,* bidirectional sugar transporter SWEET (Lus10003143)*, and *serine/threonine protein kinase (Lus10027221*,* Lus10019036).* The other subcluster comprised genes with highest expression in leaf tissue. This sub-cluster comprised 32 genes including genes encoding monooxygenase/heme binding protein (*Lus10017775*), premnaspirodiene oxygenase/heme binding protein (*Lus10030924*), dioxygenase/metal ion binding protein (*Lus10022415*), transcription regulatory nucleic acid binding protein (*Lus10005886*), auxin-activated signaling pathway gene (*Lus10042470*), Cytochrome P450 superfamily proteins (*Lus10033661*,* Lus10020360*), transmembrane transport (*Lus10013276*,* Lus10038616*), protein kinase (*Lus10019657*), serine carboxypeptidase (*Lus10012718*) etc. The other major clusters comprised the rest 55 genes, which were further grouped as per highest gene expressions in all three reproductive tissues but leaf or stem. The important genes related to flowering trait in the cluster were squamosa promoter-binding-like protein (*Lus10013999*), developmental protein SEPALLATA 1 (*Lus10034371*,* Lus10005080*), expansin (*Lus10034227*), subtilisin-like protease, SBT (*Lus10034900*), zinc finger protein CO3 (*Lus10027344*), MYB transcription factor (*Lus10019085*), cytochrome P450 family protein (*Lus10023144*) etc.

### Gene expression profile of flowering gene orthologs in floral and vegetative tissues

**Fig. 6:**
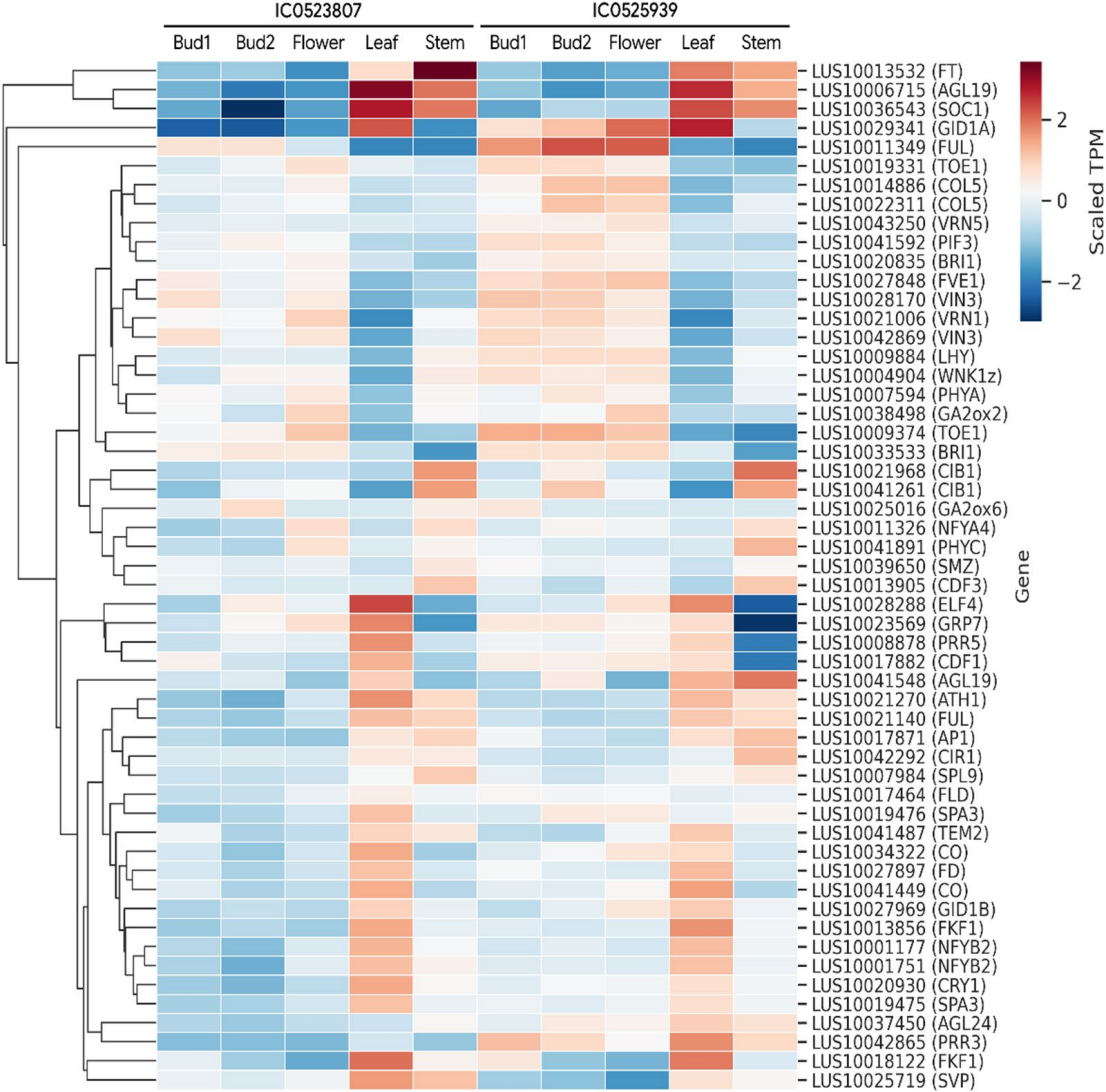
Gene expression profile of linseed orthologs of *Arabidopsis thaliana* flowering genes in linseed floral and vegetative tissues of early flowering linseed accessions IC0525939 and IC0523807. Locus ids and gene name have been shown on right side and expression-based clustering on left side. Transcript abundances in the form of TPM (Transcripts per Million) in colour gradient is shown. The gene expression profiles of orthologs of Arabidopsis flowering genes especially key flowering integrators reflect the conserved roles of these genes in the regulation of flowering in early flowering linseed accessions.

The flowering genes known in *Arabidopsis* under different pathways were searched in linseed genome for potential orthologs. From the total of 109 major flowering genes of *Arabidopsis* for which orthologs were detected in linseed, 54 (including paralogous genes) were found to be differentially expressed (Table S8). The top upregulated genes in floral tissues were *VIN3*,* FUL*,* BRI1*,* PHYA*,* PIF3*, and *TOE1* (Fig. 6). The most downregulated genes in floral tissues included *AGL19*,* SOC1*,* FT*,* CIB1*, and *SPA3*. From the photoperiod pathway, *Constans (CO)*,* Constans-Like 5 (CO5)*,* FT*,* Nuclear Factory Y subunits (NF-Ys) NFYA4*,* NFYB2*, and *PHY-A* were found differentially expressed. FT expression was highest in leaf and stem in both the accessions, whereas *CO*,* NFYB2*, showed highest expression in leaf. Under the autonomous pathway, *Short Vegetative Phase (SVP)*, and *Suppressor of Overexpression of Constans 1 (SOC1)* were found to be down regulated in floral tissues, while key genes such as *Flowering Control Locus C (FLC)* and *Flowering Control Locus A (FCA)* were not among the DEGs. In hormonal pathway, *gibberellin 2-oxidase (GA2ox2)*,* gibberellin 2-oxidase 6 (GA2ox6)*,* GA Insensitive Dwarf1B (GID1B)*,* GA Insensitive Dwarf1A (GID1A)*,* Squamosa Promoter Binding Protein-Like 9 (SPL9)* were among the DEGs.

### Expression profiling of GWAS-identified flowering time candidate genes

**Fig. 7:**
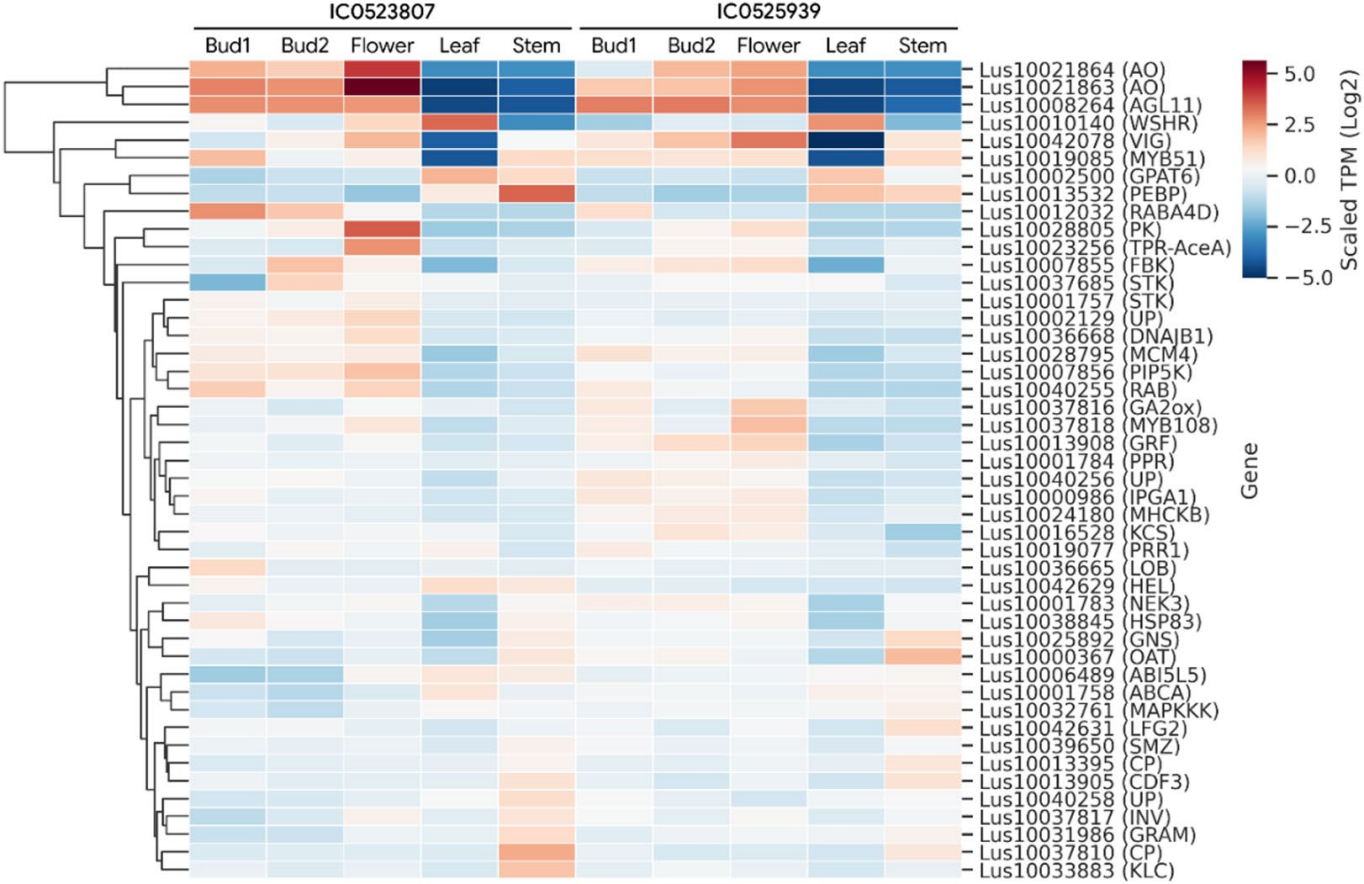
Gene expression profile of potential candidate genes for flowering time traits in early flowering linseed accessions IC0525939 and IC0523807. Locus ids have been shown on right side and expression-based clustering on left side. Transcript abundances in the form of TPM (Transcripts per Million) in colour gradient is shown. These genes were identified as common between previously reported genome-wide association studies and the differentially expressed genes observed in vegetative and reproductive tissues of the early flowering linseed accessions.

A total of 143 potential candidate genes for flowering time traits, identified from earlier GWAS studies^13,36^, were selected for gene expression analysis in the early flowering accessions (Table S9). The expression of these genes was examined across vegetative and reproductive tissues in both accessions to assess their expression patterns, tissue specificity, and to infer their roles in flowering regulation. Out of the 143 candidate genes analyzed, 46 genes were found to be differentially expressed in both accessions, indicating consistent expression changes between vegetative and reproductive tissues. The detailed expression profiles of these 46 differentially expressed genes are presented in Fig. 7. The genes with higher expression in buds and flowers included *L-ascorbate oxidase homologs (Lus10021863*,* Lus10021864)*,* Agamous-like MADS-box protein AGL11 (Lus10008264)*,* Vignain (Lus10042078)*, and *transcription factor MYB51 (Lus10019085).* Genes with highest expression in leaf comprised *FT (Lus10013532)*,* desiccation protectant protein Lea14 homolog (Lus10010140)*,* Glycerol-3-phosphate 2-O-acyltransferase (Lus10002500)*, and *Ras-related protein (Lus10012032)*, whereas genes,* peptidase S10 (Lus10037810)*,* kinesin light chain-related 1 (Lus10033883)*,* Zinc finger (Lus10040258)*,* PEBP (Phosphatidylethanolamine-binding protein) (Lus10013532)*, and* alkaline/neutral invertase C (Lus10037817)* showed higher expression in stem.

**Fig. 8:**
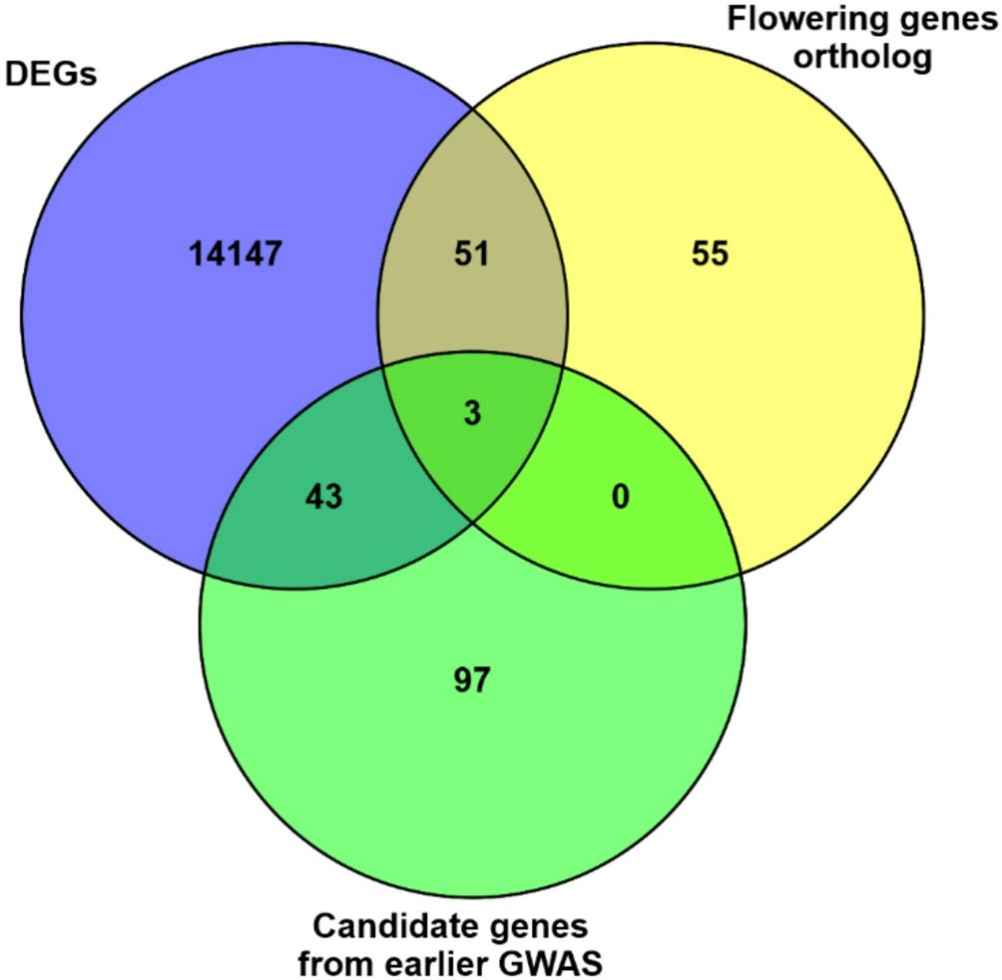
Venn diagram illustrating the overlap of promising candidate genes among three datasets, differentially expressed genes (DEGs), GWAS-derived candidate genes, and flowering gene orthologs. The intersections highlight genes that are common to two or all three datasets, representing key candidates for flowering regulation.

To identify the most promising candidate genes for flowering time traits in linseed, a comparative analysis was performed among 14,244 differentially expressed genes (DEGs) identified in this study, 109 orthologs of known flowering genes from *Arabidopsis thaliana*, and 143 potential candidate genes reported in earlier GWAS studies (Fig. 8). Of these, 54 *Arabidopsis* flowering gene orthologs and 46 GWAS-based candidate genes for flowering time in linseed were found to be differentially expressed. Comparison across the three datasets (DEGs, flowering gene orthologs, and GWAS-derived candidate genes) revealed three genes that were common to all three groups. By integrating these three complementary approaches, namely differential expression analysis, orthology with known *Arabidopsis* flowering genes, and GWAS-based candidate gene information, the most promising candidate genes for flowering time regulation in linseed were identified, which included *FT-like protein (Lus10013532)*,* AP2/ERF transcription factor* (*SMZ*) *(Lus10039650)*, and *Dof-type domain-containing protein (CDF3) (Lus10013905).*

### Whole genome resequencing of early and late flowering accessions

To unravel the allelic variation in the potential candidate genes associated with flowering in linseed, two early (IC0523807, and IC0525939) and two late flowering (EC0115148 and EC0718827) accessions were selected for whole genome resequencing. The two early accessions were the same for which transcriptome sequencing was undertaken. Total 24Gbp data of short reads were obtained and submitted in the Genbank SRA database with the BioProject accession number PRJNA1207411 (Table S10). Allelic variation for 134 key flowering genes was studied in two early and late flowering accessions (Table S11).

**Fig. 9:**
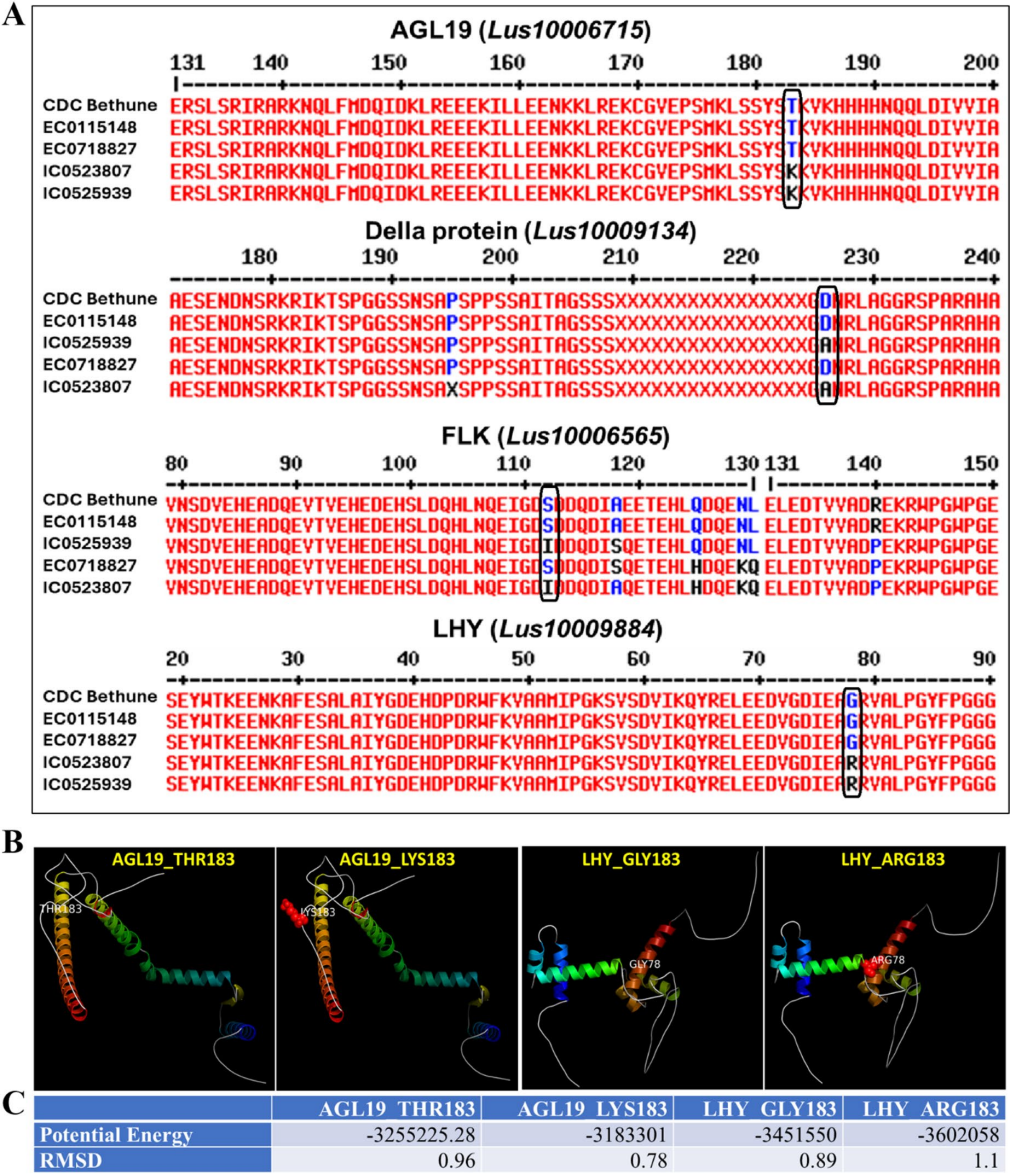
Allelic variation in key flowering genes, AGAMOUS-like 19 (AGL19), Della protein, Target of EAT1 (TOE1), and Late Elongated Hypocotyl (LHY) in linseed. A: Multiple sequence alignment of deduced protein sequences based on nucleotide variations in the early (IC0525939, IC0523807) and late genotypes (EC0115148, EC0718827) with CDC Bethune as reference. The box showed crucial amino acid variation between early and late genotypes. B: Homology model based 3-D protein structure of AGL19 and LHY with single amino acid substitutions. C: Effect of single amino acid substitution on potential energy and RMSD (Root Mean Square Deviation) values of the proteins. The lower potential energy and RMSD are indicative of better stability and conformation.

Several distinctive patterns of variants were observed in a few important genes related to flowering, such as *AGAMOUS-like 19 (AGL19) (Lus10006715)*,* della protein (Lus10009134)*,* flowering locus K (FLK) (Lus10006565)*,* Target of EAT1 (TOE1) (Lus10019331)*, and *Late Elongated Hypocotyl (LHY) (Lus10009884)* (Table 2). The linseed *AGL19 (Lus10006715)*, a 3.5 kb gene harboured 52 SNPs/indels, of which 47 were in intron regions and 3 SNPs were in exons. Of the exonic 3 SNPs, only one was non-synonymous affecting change in amino acid threonine (T) to lysine (K) at position 183 (T183K) (Fig. 9A). Both early flowering accessions IC0523807, and IC0525939 showed threonine, whereas both the late flowering accessions (EC0115148 and EC0718827) showed lysine at position 183.

**Table 2:** Gene haplotype in early (IC0523807, IC0525939) and late (EC0115148, EC0718827) flowering linseed accessions.

SNP Position	CDC Bethune	EC0115148	EC0718827	IC0523807	IC0525939
***AGL19- Lus10006715***					
387	c	g	c	c	c
396	g	a	g	g	g
553	a	g	a	a	a
563	a	g	a	a	a
576	g	a	g	g	g
1155	g	g	g	c	c
1161	c	c	c	t	t
1179	t	t	t	c	c
1277	a	a	g	g	g
1310	t	t	c	c	c
1327	a	a	-	-	-
1355	t	t	c	c	c
1377	t	t	c	c	c
1378	g	g	t	t	t
1404	t	t	c	c	c
1414	c	t	t	t	t
1452	t	t	g	g	g
1458	a	a	g	g	g
1460	t	t	c	c	c
1660	g	g	a	g	a
1707	t	t	t	c	c
1726	t	t	t	g	g
1728	a	a	a	g	g
1761	a	a	a	g	g
1777	c	c	c	a	a
1785	a	a	a	g	g
1788	g	g	g	a	a
1817	a	a	a	c	c
1911	a	a	a	t	t
2069	a	a	a	c	c
2092	c	c	c	t	t
2099	t	t	t	a	a
2162	g	g	g	a	a
2210	t	t	t	c	c
2211	g	g	g	t	t
2225	a	a	a	g	g
2305	g	g	g	a	a
2308	g	g	g	t	t
2360	t	t	t	c	c
2407	a	a	a	g	a
2527	A	A	A	G	G
2687	-	-	-	a	a
2873	c	c	c	t	t
2874	g	c	c	g	g
2912	g	g	a	g	g
3014	c	t	t	c	c
3074	t	c	c	t	t
3237	t	t	t	t	a
3277	a	a	a	-	c
3278	a	a	a	-	a
3279	t	t	t	-	t
3280	c	c	c	-	c
3281	a	a	a	-	a
3282	a	a	a	-	a
3301	t	t	t	t	c
3358	C	C	C	A	A
3524	T	T	T	T	A
***Della protein Lus10009134***					
677	A	A	A	C	C
693	G	G	A	A	A
***FLK Lus10006565***					
335	G	G	G	T	T
339	C	C	C	T	T
351	A	A	T	A	T
352	G	G	T	G	T
355	G	G	C	C	C
375	G	G	T	T	G
387	T	T	A	A	T
389	T	T	A	A	T
419	G	G	C	C	C
1013	g	c	g	g	g
1020	t	a	t	t	t
1035	t	g	t	t	t
1053	a	c	a	a	c
1058	c	a	c	a	a
1085	t	a	t	a	a
1089	c	a	c	a	a
1096	g	a	a	a	a
1133	a	g	g	g	g
1141	g	t	t	t	t
1341	c	g	g	g	g
1354	t	g	g	g	g
1380	a	g	g	g	g
1385	a	a	a	g	a
1391	c	t	t	t	t
1392	t	c	c	c	c
1397	c	a	a	a	a
1409	a	g	g	g	g
1425	a	g	g	g	g
1455	a	a	a	c	c
1457	g	g	g	a	a
1477	a	a	a	t	t
1478	g	g	g	a	a
***TOE1 Lus10019331***					
1058	G	T	G	G	G
1128	G	C	G	G	G
1150	c	a	c	c	c
1151	g	t	g	g	g
1205	t	-	t	t	t
1238	T	C	T	T	T
1262	C	T	C	C	C
1930	a	c	c	a	a
2135	-	a	a	-	-
2152	-	g	g	-	-
2153	-	c	c	-	-
2154	-	a	a	-	-
2155	-	g	g	-	-
2156	-	a	a	-	-
2157	-	g	g	-	-
2158	-	g	g	-	-
2159	-	g	g	-	-
2160	-	g	g	-	-
2161	-	g	g	-	-
2162	-	g	g	-	-
2163	-	c	c	-	-
2164	-	g	g	-	-
2165	-	t	t	-	-
2303	a	g	g	a	a
2578	C	C	-	C	C
2579	A	A	-	A	A
2580	A	A	-	A	A
2581	C	C	-	C	C
2582	A	A	-	A	A
2583	A	A	-	A	A
2584	C	C	-	C	C
2585	A	A	-	A	A
2586	A	A	-	A	A
***LHY Lus10009884***					
232	G	G	G	C	C
577	T	T	T	C	C

SNP position is with reference to CDC Bethune. Small and capital letters of nucleotides bases indicate intronic and exonic SNPs, respectively. Hyphen (-) indicates deletion at respective position.

The *Della protein (Lus10009134)* showed two SNPs, of which SNP at position 677 was non-synonymous resulting in change in amino acid aspartic acid (D) in both the late accessions to alanine (A) in early flowering accessions (Fig. 9A). *FLK (Lus1000656532)*, a 4.2 kb gene harboured total 32 SNPs, of which 23 and 9 were in intronic and exonic regions, respectively. From the nine exonic SNPs, six were nonsynonymous resulting change in amino acids, however all but one showed distinct pattern in early and late accessions. Late flowering accessions showed serine (S) at 112th position, whereas in both the early accessions it was replaced with isoleucine (I) (Fig. 9A).

*TOE1 (Lus10019331)*, 2.7 kb gene showed 9 SNPs, of which 4 and 5 were in exonic and intronic regions, respectively. Two SNPs were nonsynonymous, however with no clear pattern in early and late accessions. Further, there were two indels in the gene, both in the 9^th^ intron (Table 2). A 15 bp insertion was observed in both the late flowering accessions, EC0115148 and EC0718827. A deletion of 9 bp was also observed in one of the late flowering accession EC0718827.

*LHY (Lus10009884)* gene harboured two SNPs at 232nd and 577th position in the first and second exons, respectively. A SNP at 232nd position was nonsynonymous in nature which changed amino acid glycine (G) to arginine (R) at 78th position in the protein. Both the late and early flowering accessions showed distinct pattern with glycine in late and arginine in early accessions (Fig. 9A).

To study effect of single amino acid change in proteins of early and late accessions, 3-D structure of AGL19 and LHY were constructed with both the variant amino acids (Fig. 9B, C). AGL19_Thr183 (variant in late accessions) showed lower potential energy compared to AGL19_Lys183 (variant in early accessions), indicating greater structural stability and a more favourable conformation in the former. Despite this, both structures were very similar, as suggested by their RMSD (Root Mean Square Deviation) values. In the case of LHY, the lower potential energy of LHY_Arg183 (variant in early accessions) indicates better stability and conformation than LHY_Gly183 (variant in late accessions), though the structural deviation was only moderate.

## Discussion

Flowering time in linseed is a critical component in India as well as in the global context to expand the area of cultivation, circumvent the abiotic stress (frost, drought, terminal heat etc.) and to maximize the yield advantage and commerce. There is substantial variation in flowering time in linseed among the germplasm accessions from different agro-ecological and geographical locations^32,58,59^. Unlike the model plant and other major crop plants, the understanding on the genetic architecture of flowering time in linseed is in the nascent stage as only a few comprehensive studies have been reported using QTL mapping/GWAS/ genome-wide variation analyses^13,33,34,36,60^. Other crucial complementary approaches such as transcriptomics to understand the flowering regulation are far more limited. Recently, House et al.^11^ gave a comprehensive overview of transcriptome profile of shoot apical meristem (SAM) at four different stages of a single flax cultivar ‘Royal’, focussing especially on the flowering gene homologs from *Arabidopsis*. The study identified DEGs encompassing major flowering pathways, enriched transcription factor families (*MADS-box*,* SBP*) and major regulators such as SOC1, FUL, and AP1. Although, SAM constitute crucial site of floral transition, the perception of internal (hormones) and external (photoperiod) cues, and generation of flowering signal occurs at leaves and transported via phloem/stem. In our study, two early flowering accessions were used to study the transcriptomics profile in floral bud tissues at the two developmental stages, flower, leaf, and stem. The aim was to capture the wide spectrum of genes and gene expression dynamics involved in floral development and flowering regulation in linseed. Leaf and stem have been included in the transcriptome study as these sites are crucial in perception, generation, coordination, and transport of flower inducing signals to shoot apex to initiate flowering^61,62^. The differential expressions of genes were identified by comparing each floral developmental stage (bud1, bud2, flower) against leaves and stem in each accession. Comparison of number of DEGs in two accessions showed near similar upregulated and downregulated genes with a few exceptions. In both the accessions IC0523807, and IC0525939, the highest number of upregulated DEGs, 2081 and 2268, respectively were under combination ‘bud1 vs. leaf, bud2 vs. leaf, flower vs. leaf’ tissues (Fig. 3) suggesting these genes could be involved in floral organ developments and maintenance. The biological replicates of the studied tissues were coherent, as evidenced by PCA clustering (Fig. 2) and pairwise correlation analysis of the top 100 DEGs, Arabidopsis ortholog DEGs, and GWAS-derived candidate genes, which showed high concordance in expression profiles between replicates (*r* > 0.93) (Fig. S7).The top 20 enriched KEGG terms in DEGs from the KEGG pathway analysis included MYB family transcription factor, peroxidase, cytochrome P450 family 1 subfamily A1, and calcium-binding protein CML. MYB transcription factors have been shown to play important roles in floral development and flowering time^63^. Peroxidase, and cytochrome P450 family proteins among the enriched KEGG were as expected. Peroxidase activities have long been considered as an indicator of initiation of floral induction and therefore have been used as biochemical markers to characterize the flowering induction phase^64^. Cytochrome P450 family proteins are known to play a diverse role in plants and also regulate plant hormone metabolism which in turn control cell division and expansion, flower and floral pigments formation, vascular differentiation, fruit growth etc.^65^. Similarly, MADS box transcription factors have been known to be associated with plant growth and development including floral organ development, flowering time regulation, and female, male gametophyte development^66^. The enriched pathways included histone H2A (K11251), histone H4 (K11254), histone H3 (K11253), E3 ubiquitin-protein ligase (K19041, K11982, and K22378), auxin-responsive protein IAA (K14484), aquaporin PIP (K09872), brassinosteroid 6-oxygenase (K09590), and WRKY transcription factor (K13424), which have been shown to have role in floral development and/or flowering time regulation^67–72^.

The circadian clock in plant consists of three main modules, the input pathway, the central oscillator, and the output pathway^73^. From the input pathway, in this study far-red-rich light photoreceptors- *PHY-A* and *blue/UV-A light-receptor cryptochrome*-* CRY1* were found differentially expressed (Fig. 4, Fig. 6). PHY-A under far-red-rich light promotes flowering in Arabidopsis and act as positive regulator of CO^74^. The cryptochromes are well known blue light receptors and have diverse functions in plants including stem elongation, flowering time, stomatal opening, circadian clock etc.^75^. CRY1 compared to the CRY2 were found to have milder effect on flowering, which could be photoperiod independent, however, it may have a temperature-dependent activity in flowering-time regulation^73,76^. From the central oscillator of circadian clock, *LHY*, and *TOC1* were among the DEGs (Fig. 4, Fig. 6). It has been shown that LHY similar to TOC1 regulates photoperiodic flowering *via* the circadian clock as the *LHY* mutants exhibit accelerated flowering in Arabidopsis^77^. From the output pathway, *CO* and *FT* were found to be differentially expressed. The other important components although could have expression in the studied tissues, however, not DEGs.

Plant hormones are known to play an important role regulating flowering time through the photoperiod pathway^78^. In present study, under the hormone pathway, the highest number of DEGs were identified for ABA (236), followed by GA (58), BR (11), auxins (6), and cytokinin (5) (Fig S5). Role of abscisic acid (ABA) is well known in drought, osmotic, and salinity stress^18^. However, there has been growing number of evidence unravelling involvement of ABA in plant growth and development, such as hydrotropism, xylem formation, and leaf initiation as well as development, and floral transition^79^. ABA signalling influences photoperiodic flowering by interacting with key flowering regulators and generation of FT in leaves^79^. Under long-day conditions, ABA modulates flowering by fine-tuning *CO*, *FT*, and *SOC1* expression. ABA signalling mutants show flowering defects linked to reduced *FT* expression. ABFs promote *SOC1*, which indirectly enhances *FT* expression by suppressing repressors like *TEM1/2* and *TOE1*. Additionally, NF-Y transcription factors integrate ABA and flowering signals, adding complexity to this regulatory network^79^. The roles of GAs, among the phytohormones are particularly well established in *Arabidopsis* flowering. Several studies have demonstrated that exogenous application of GA, overexpression of the biosynthetic gene (*GA5*), and the GA signalling promoted flowering^78^. It has been shown that GA induces FT expression by a CO-dependent pathway. The DELLA protein, the prominent repressor of GA signalling physically interacts with CO to inhibit CO/FT-mediated flowering under LD, suggesting the synergistic modulation of flowering under LDs by GA and photoperiod signalling^80^.

There have been substantial number of DEGs found to be involved in redox homeostasis in present study (Fig S5). The cellular redox state plays a vital role in regulating metabolism, signalling, and overall function. It has been earlier shown that the circadian clock regulates ROS homeostasis, and the ROS signals impact other metabolic processes regulated by the circadian clock^81^. There also exists association between glutathione accumulation and floral development and pollen germination^82^.

Among the top 100 DEGs, those with higher expression in stem included mostly transporters such as *nitrate and peptide transporter family (NPF) (Lus10039521)*,* proton-dependent oligopeptide transporter (Lus10007437)*,* metal transporter (Lus10024069)*,* ABC transporter B family member 1 (Lus10014427)*,* bidirectional sugar transporter SWEET*, and *serine/threonine protein kinases*. Among them, NPF, sugar transporter SWEET, and S/T protein kinases show direct or indirect association with flowering regulation due to their roles in nutrient, and sugar pathways and through their interaction and/or intersection with the flowering pathways. In particular, Nitrate Transporter 1.1 (NRT1.1) affects flowering by influencing *FT* gene expression with its interaction with the *FLC*-dependent flowering pathway^83^. NRT1.1 is also thought to be involved in other signalling pathways that attenuate *FLC* expression, albeit in a nitrate-independent manner. The bidirectional sucrose transporter, SWEET10 was found to act downstream of FT during floral transition of *Arabidopsis*^84^. Further, overexpression of *SWEET10* leads to higher expression of flowering time genes and early flowering *per se*. Potential roles of MAPKs have been highlighted in floral growth and development specifically including pollen, anther, and gametophyte development^85^. From the gene cluster with highest expression in leaf comprised total 32 genes, which included, monooxygenase/heme binding protein, cytochrome P450 superfamily proteins, protein kinase etc. These genes may not have direct relation with floral organogenesis or flowering regulation; however, it could have indirect role *via* hormonal biosynthetic pathways, flowering pathways, signalling pathways such as auxin-activated signalling pathways, protein kinases signalling etc.^85^. There were 55 genes among the top 100 DEGs, with higher expression in floral tissues. The list comprised genes encoding squamosa promoter-binding-like protein (*Lus10013999*), developmental protein SEPALLATA 1 (*Lus10034371*,* Lus10005080*), expansin (*Lus10034227*), zinc finger protein CO like (*Lus10027344*), and MYB transcription factor (*Lus10019085*) which are known to have role in floral development and / or flowering time regulation^86–89^.

*Arabidopsis thaliana*, besides being a model plant with well-studied flowering regulation pathways, is also a facultative long-day plant, similar to linseed, which flowers more quickly under long-day conditions but can still flower under short-day conditions, although at a slower rate^5^. This makes *Arabidopsis* a relevant model to compare with, and to study the flowering regulation in linseed. To leverage advancements in the understanding of flowering regulation in *Arabidopsis*, the known flowering genes from *Arabidopsis* were used to identify their potential orthologs in linseed and to analyse their expression in the studied early-flowering accession of linseed. In this context, orthologs of major *Arabidopsis* flowering genes were identified in linseed, and their gene expression was analyzed in the studied tissues. Of the 109 potential orthologs, 54 were differentially expressed (Fig. 6, Table S8). The differentially expressed orthologs included known genes including *VIN3*,* FUL*,* BRI1*,* PIF3*,* TOE1*,* AGL19*,* SOC1*,* CIB1*,* SPA3*, along with known genes from photoperiod pathway (*CO*,* COL5*,* FT*,* NFYA4*,* NFYB2*,* PHY-A*), autonomous pathway (*SVP* and *SOC1*), and hormonal pathway (*GA2ox2*,* GA2ox6*,* GID1B*,* GID1A*,* SPL9*). A similar approach was followed in the flax cultivar ‘Royal’ flax and studied gene expressions of 722 putative flax flowering genes, which also included the multiple homologues for a single *Arabidopsis* flowering gene^11^. The gene expression of 80% of these genes were observed in the studied tissues, and 30% of them were differentially expressed.

In linseed, a few recent studies using genome wide association studies have identified genomic regions in the form of quantitative trait nucleotides (QTNs) and potential candidate genes for flowering time^13,36^. In present study the gene expression of those potential candidate genes was also studied and identified 46 differentially expressed genes further highlighting their importance in linseed flowering. The present study has included later stages of floral development along with leaf and stem tissues, whereas earlier study^11^ focused on the earlier stages, shoot apical meristem (SAM). If DEGs from our study were also differentially expressed at the SAM of ‘Royal’ flax, a comparison was made between the top 100 DEGs (Fig. 5), differentially expressed flowering gene orthologs (Fig. 6), and GWAS-derived candidate genes (Fig. 7) and identified 56, 27, and 16 genes, respectively, that overlapped with DEGs reported in the SAM of the cultivar ‘Royal’^11^ (Table S12). Although caution needs to be exercised due to differences in genetic background, environment, and tissue type, this substantial overlap suggests that our study captured a considerable portion of potential early floral signals. Beside the known flowering genes, a few genes could be potential candidates such as *Lus10042564* and *Lus10037817* encoding an unknown protein, and alkaline/neutral invertase, respectively. Lus10042564 was identified candidate gene in earlier GWAS^36^, and was among the DEGs in the present study. Interestingly, this gene was also among the DEGs in SAM of the flax cultivar ‘Royal’ for flowering time study and also identified as novel or linseed lineage-specific gene^11^. The conserved domain search showed the protein harbours CPP1-like (CHAPERONE-LIKE PROTEIN OF POR1-like) (pfam11833) and DnaJ domain or J-domain (cd06257) with probable function in protein translation, folding, unfolding, translocation, and degradation. The other potential candidate Lus10037817 (alkaline/neutral invertase) was also identified as candidate in earlier GWAS^36^ and among the DEGs in present study as well as in House et al.^11^. The gene ontology of the protein showed biological role in the regulation of timing of meristematic phase transition (GO:0048506) and circadian rhythm (GO:0007623). Alkaline/neutral invertases have been shown to play role in growth and development including flowering regulation and circadian clock in Arabidopsis^90,91^.

To avoid identifying DEGs that reflect tissue-specific expression related to development and function rather than flowering regulation, an additional approach integrating GWAS-identified candidate genes and known flowering genes from well-characterized flowering pathways of Arabidopsis was employed to narrow down the key flowering candidate genes (Fig. 8). This led to the identification of three genes, *FT (Lus10013532)*,* AP2/ERF transcription factor SCHLAFMÜTZE* (*SMZ*) *(Lus10039650)*, and CDF3 (*Dof-type domain-containing protein) (Lus10013905)* as the most promising candidates. Role of FT is well established in plants. It encodes a protein that plays a crucial role in the early stages of angiosperm flowering, regulates a complex signalling network and facilitates differentiation of apical meristems into flowers^21,92^. Its higher expression in the leaves of both accessions (Fig. 6 and 7) is consistent with the well-established mechanism of FT-mediated floral induction, wherein FT is expressed in leaves and the FT globular protein is translocated to the shoot apical meristem (SAM) via the phloem^93^. Lus10013532 has also been reported as an FT homolog and shown to be expressed in the shoot apical meristem of early-flowering linseed lines^9^. FT acts as a central integrator of multiple flowering pathways, coordinating signals from numerous genes involved in the regulation of floral transition in *Arabidopsis*^94–96^. Therefore, activation of FT-mediated flowering in the studied early flowering linseed accessions may occur through the photoperiod and circadian pathways (as expression of both FT and its positive regulator CO homologues are high in leaves), the autonomous pathway (lower level of FLC in all the studied tissues in-spite no vernalization like prolonged cold conditions), and / or the gibberellin (GA) pathway (higher expression of GID1 homologous in leaves).

The second crucial candidate gene, SMZ, an AP2/ERF transcription factor, is an ortholog of a well-characterized flowering repressor in *Arabidopsis*. SMZ has been shown to directly bind to the promoter region of FT as well as to other flowering regulators acting both upstream and downstream of FT^94^. In addition, SMZ is a known target of the regulatory microRNA miR172, which plays a key role in flowering-time regulation. In soybean, a functionally similar AP2/ERF transcription factor, TOE4b, has been identified through GWAS and shown to bind to the promoters of key flowering integrator genes FT2a and FT5a, thereby repressing their transcription^97^. Interestingly, a specific haplotype of TOE4b has been reported to confer early flowering. In agreement with this regulatory model, SMZ (*Lus10039650*) expression was low across all the studied tissues including leaves, with only slight elevated expression in stem (Figs. 6 and 7), suggesting a similar mechanism of SMZ mediated regulation of FT in linseed.

With respect to the third promising candidate gene *DOF (CDF3)*, several of them have been shown to delay flowering by repressing *CO* transcription and combined mutations in a few of these *DOF*s were resulted in photoperiod-insensitive early flowering in *Arabidopsis*^98^. Overexpression of *DOF* genes from plants such as oilseed rape, and medicago resulted in delayed flowering^99,100^. Expression of *CDF1* was found low in leaf tissues of both the accession, while expression of *CO* high (Fig. 6) similar in line with the earlier studies.

Allelic variation studied using whole genome resequencing have revealed that for the three promising genes encoding *FT (Lus10013532)*,* AP2/ERF transcription factor (Lus10039650)*, and* CDF3 (Lus10013905)* there was no allelic variation, at least in the studied accessions. In other plants, higher expression of *FT* has been reported to promote early flowering^101^. In domesticated chickpea, a strong genetic association was found between early flowering and elevated expression of a cluster of *FT* genes compared with wild chickpea species, possibly due to *cis*-acting changes leading to deregulated expression of this gene cluster^102^. In linseed, further studies involving promoter analysis and gene expression profiling of early and late-flowering accessions in a larger panel are expected to shed light on the contribution of *FT*,* AP2/ERF*, and *CDF3 per se* to early flowering.

The allelic variation of other important flowering related genes was studied in two early and two late flowering accessions through whole genome resequencing. Interesting variations have been observed in other key genes such as *AGAMOUS-like 19*,* della protein*,* FLK*,* Target Of EAT1*, and *Late Elongated Hypocotyl* (Table 2; Fig. 9). AGL19 is a MADS-box transcription factor that acts as a floral activator and functions mainly through an FLC-independent vernalization pathway. Variation in its expression and regulation plays an important role in local adaptation to different climatic conditions^103^. Ectopic expression of *AGL19* has been shown to strongly accelerate flowering, and *agl19* mutants display a reduced response to vernalization in *Arabidopsis*. Interestingly, early-flowering linseed accessions showed higher expression of AGL19 in leaf and stem tissues, suggesting that it may contribute to the promotion of flowering under local environmental conditions. Further, AGL19 has been reported to function not only under vernalization but also under non-inductive short-day conditions^104^. It is therefore possible that a similar mechanism operates in linseed, where AGL19 may play a role in flowering promotion in early flowering accessions. Non-synonymous SNPs causing distinct amino acid in early and late accessions are of interest. In *AGL19*, an amino acid threonine in late accessions is substituted with lysine at 183rd position (T183K) in early accessions, causing loss of putative phosphorylation site in early accessions (data not shown). The identified AGL19 variant (T183K) is located near the K-box region, which is important for specific protein–protein interactions^105^. The *Arabidopsis* AGL19 protein shows at least 25 amino acid variants, including an ‘S > T’ substitution at position 186; however, this specific alteration has not been associated with any reported phenotypic effect. Other AGL proteins are also known to be regulated through post-translational modifications, such as phosphorylation^106^. Therefore, functional validation of this allelic variation in linseed will be required to ascertain its effect on flowering-related phenotypes. Similarly, it is intriguing to observe distinct non-synonymous SNPs in early- and late-flowering accessions resulting in amino acid substitutions in the DELLA protein (D677A) and LHY (G78R). Based on computational protein structure analysis, LHY variant in early accessions (LHY_Arg183), showed better stability and conformation than the variant in late accessions. The allelic variations observed in these key flowering genes between early- and late-flowering genotypes suggest their potential role in regulating flowering time in linseed as single amino acid change also may have strong effect on protein structure, folding energy, stability and downstream function^107–110^. Furthermore, these variations in protein sequence may alter the strength and specificity of the corresponding protein–protein or protein–DNA interactions, potentially resulting in altered phenotypes as reported in other biological systems^111^. However, these speculations would require experimental validation.

Variations identified in the candidate genes (Table S11) across four accessions, when cross-validated using a larger association panel of 131 accessions from our previous study, showed that variation in at least three genes could be mapped and validated. These three SNPs were significantly associated with flowering time (days to 5% / 50% / 95% flowering)^36^. The associated genes included *Lus10023476 (Adenylate Kinase 1*, role in shoot system development; SNP position: *Lu07_3538758)*,* Lus10007230 (unknown protein*; SNP position: *Lu10_5001635*), and *Lus10032761 (Mitogen-activated Protein Kinase Kinase 5*, role in MAPK signaling; SNP position: *Lu10_11674762*). The significant association of allelic variation in these genes with flowering time trait in a larger panel accessions underscore importance of these loci in flowering time regulation in linseed. However, the distinctive allelic variations identified in the present study for *AGL19*,* DELLA protein*,* FLK*,* TOE1*, and *LHY* could not be detected in the GBS panel. This could possibly be due to the reduced-representation sequencing (GBS) approach used in GWAS^36^, which covers only a limited portion of the genome. Consequently, some accessions and specific genomic regions (genes) with low read depth or insufficient coverage might have been excluded from the analysis.

The allelic variants of a few important flowering genes identified in the present study represent an important advance in linseed; however, since the study was based on a small panel of accessions, validation in a larger panel is required for robust confirmation and application. Additionally, genome editing tools could be employed for functional validation of these genes or variants, enabling their further utilization in breeding programs. Overall, this study provides valuable insights and a promising direction for unravelling the complex genetic architecture underlying flowering regulation in linseed.

## Conclusions

This study, by unravelling gene expression patterns in floral and vegetative tissues of linseed, provides insights into transcriptional regulation during floral development. It highlights the complex interplay of multiple pathways, including circadian/photoperiod, hormone signalling, and sugar metabolism, and the candidate genes involved in these processes. By combining a multifaceted approach involving differentially expressed genes, comparative genomics of known flowering genes, and candidate genes from association studies in linseed, potential key regulators have been identified. Furthermore, allelic variation between early- and late-flowering accessions, explored through whole-genome sequencing of four accessions, enabled the identification of variants in important flowering genes. Overall, this study advances our understanding of flowering regulation in linseed/flax- an important trait for expanding cultivation and maximizing yield potential across diverse agro-climatic regions.

## Supplementary Information

Below is the link to the electronic supplementary material.

Supplementary Material 1

Supplementary Material 2

Supplementary Material 3

Supplementary Material 4

Supplementary Material 5

Supplementary Material 6

Supplementary Material 7

Supplementary Material 8

Supplementary Material 9

Supplementary Material 10

Supplementary Material 11

Supplementary Material 12

Supplementary Material 13

## Data Availability

The datasets generated during the current study are available in the GenBank, NCBI repository, with Bio Project accession number, PRJNA773597, PRJNA1207411.
