# Supplementary material for: Integrative transcriptome and genome resequencing reveals conserved flowering regulators and allelic variants in early- and late-flowering linseed (Linum usitatissimum L.) accessions
Source: Sci Rep. 2026 Mar 2;16:11526. doi: 10.1038/s41598-026-40729-7 (PMC13056945; doi:10.1038/s41598-026-40729-7)
Supplement: Supplementary file 3 — Supplementary Material 3 [file 41598_2026_40729_MOESM3_ESM.docx]

**Table S10**: Number of bases (Gbp) in whole genome resequenced accessions

| Accessions | R1 | R2 |
| --- | --- | --- |
| EC0115148 | 2.5 | 2.5 |
| EC0718827 | 3.1 | 3.1 |
| IC0523807 | 3.4 | 3.4 |
| IC0525939 | 3.0 | 3.0 |
