# Supplementary material for: Integrative transcriptome and genome resequencing reveals conserved flowering regulators and allelic variants in early- and late-flowering linseed (Linum usitatissimum L.) accessions
Source: Sci Rep. 2026 Mar 2;16:11526. doi: 10.1038/s41598-026-40729-7 (PMC13056945; doi:10.1038/s41598-026-40729-7)
Supplement: Supplementary file 13 — Supplementary Material 13 [file 41598_2026_40729_MOESM13_ESM.docx]

**
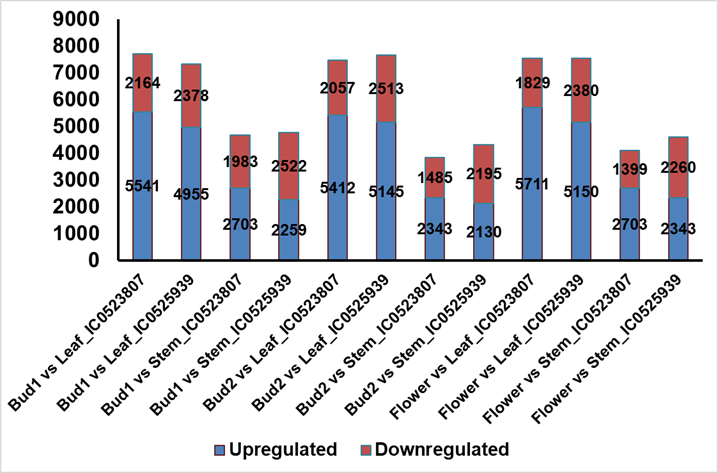
Fig. S1 Summary of differentially expressed genes (up- and down regulated) in different combination of reproductive** *vs*. **vegetative tissues in two linseed accessions IC0525939 and IC0523807.**

**Supplementary Figure S2a: Volcano plot depicting DEGs in comparison ‘Bud1 *vs* Leaf’ in accession IC0523807**

**
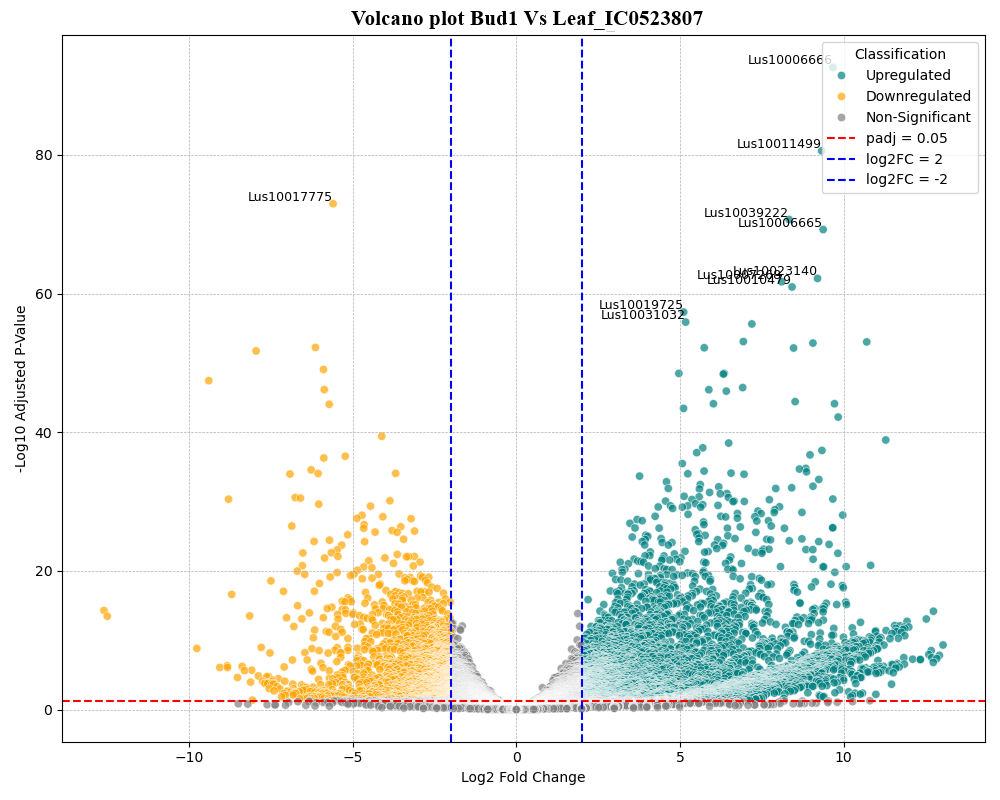
**

**Supplementary Figure S1b: Volcano plot depicting DEGs in comparison ‘Bud2 *vs* Leaf’ in accession IC0523807
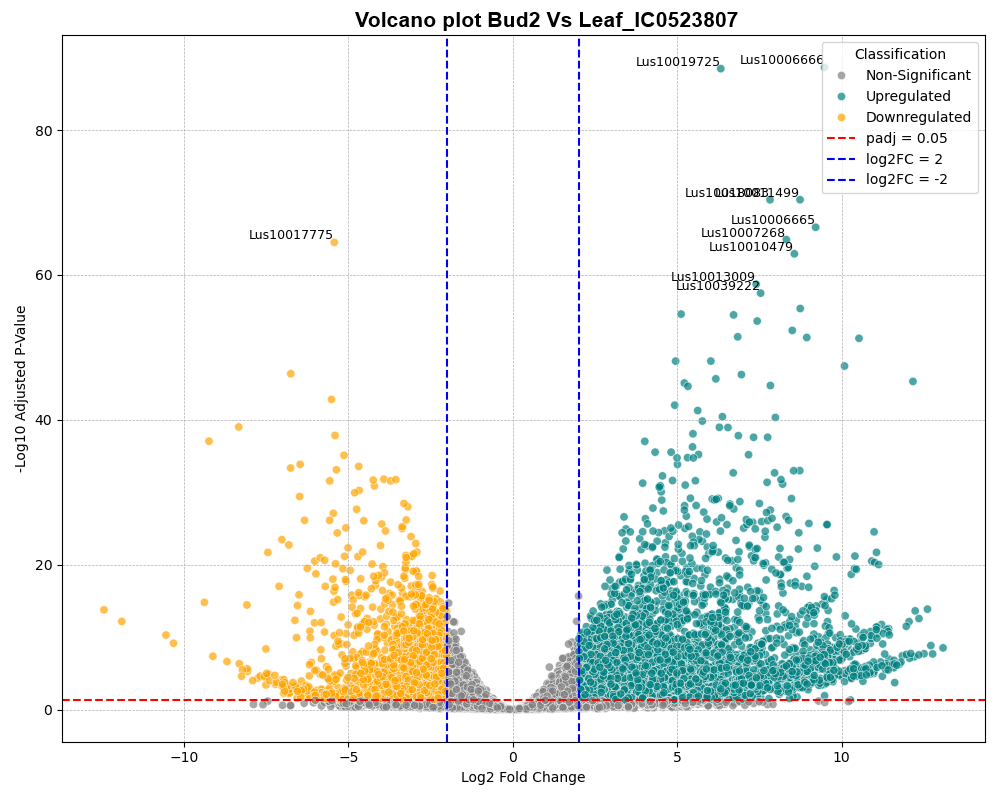
**

**Supplementary Figure S2c: Volcano plot depicting DEGs in comparison ‘Flower *vs* Leaf’ in accession IC0523807**

**
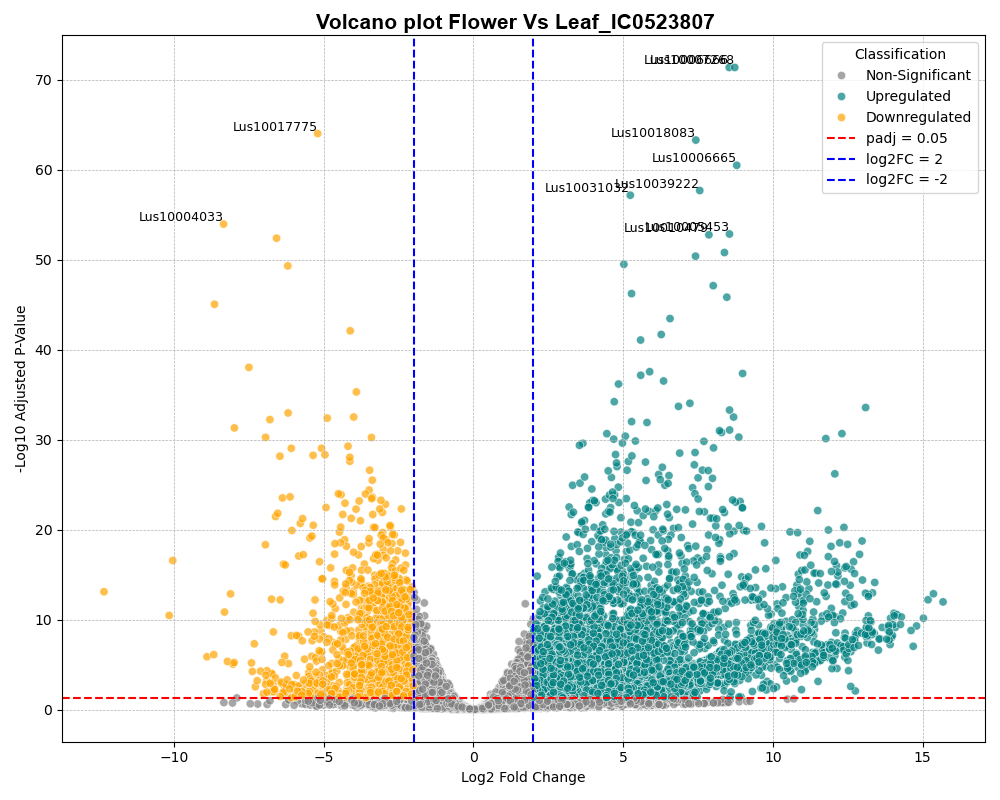
**

**Supplementary Figure S2d: Volcano plot depicting DEGs in comparison ‘Bud1 *vs* Stem’ in accession IC0523807
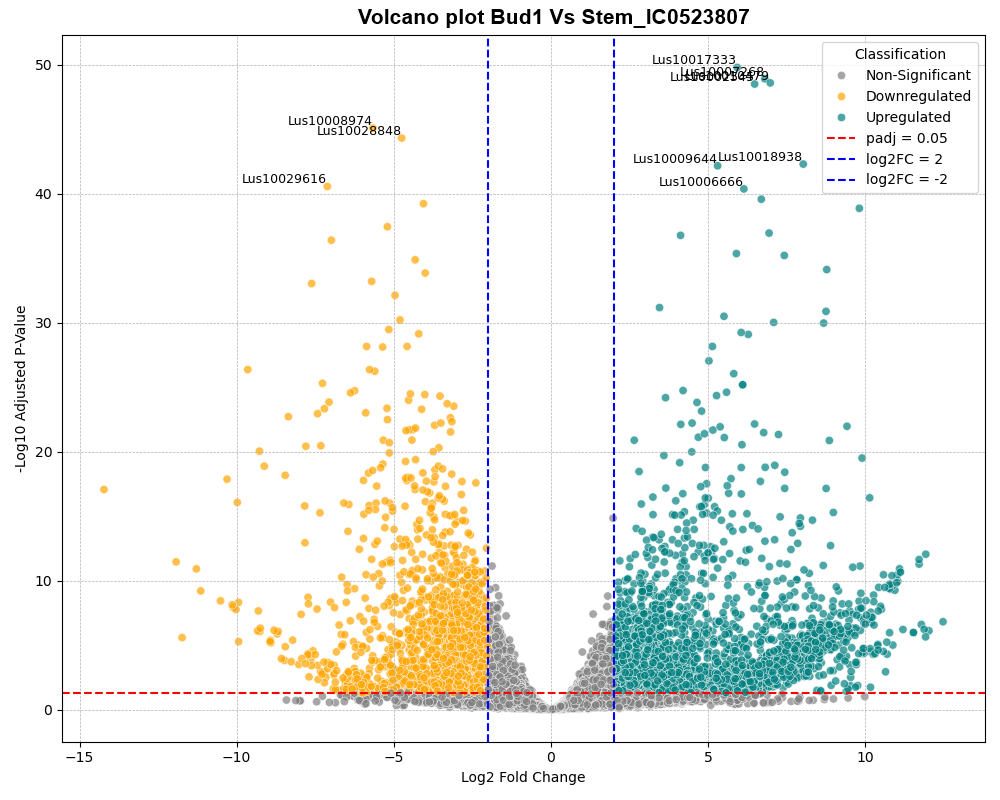
**

**Supplementary Figure S2e: Volcano plot depicting DEGs in comparison ‘Bud2 *vs* Stem’ in accession IC0523807**

**
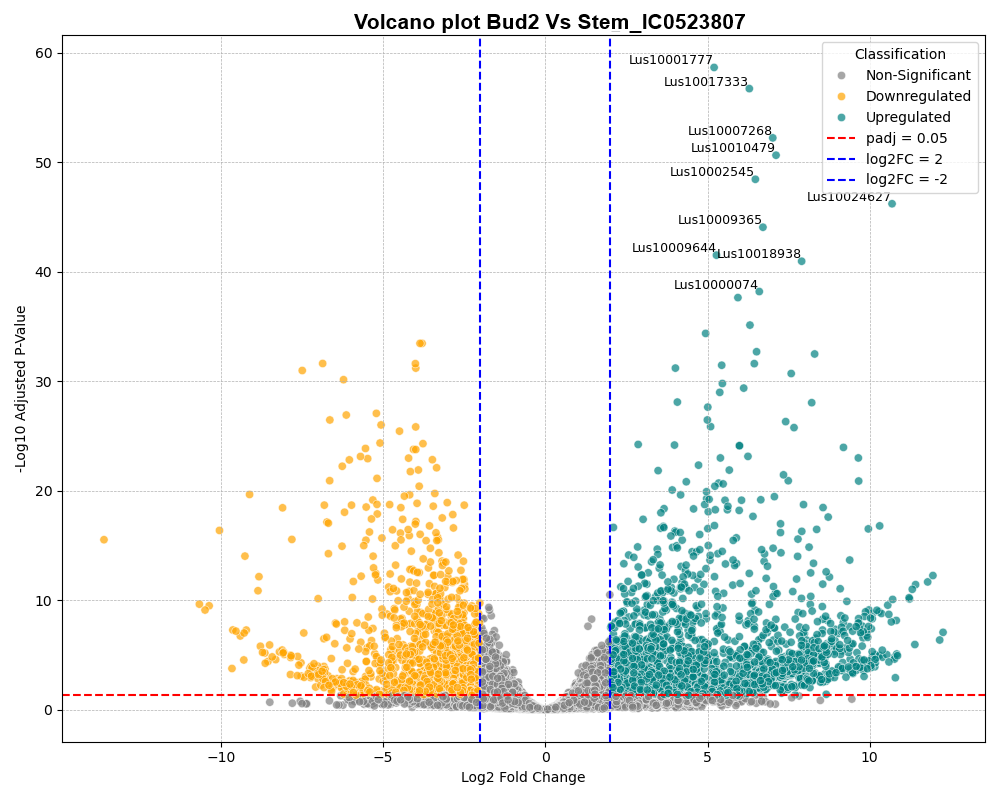
**

**Supplementary Figure S2f: Volcano plot depicting DEGs in comparison ‘Flower *vs* Stem’ in accession IC0523807
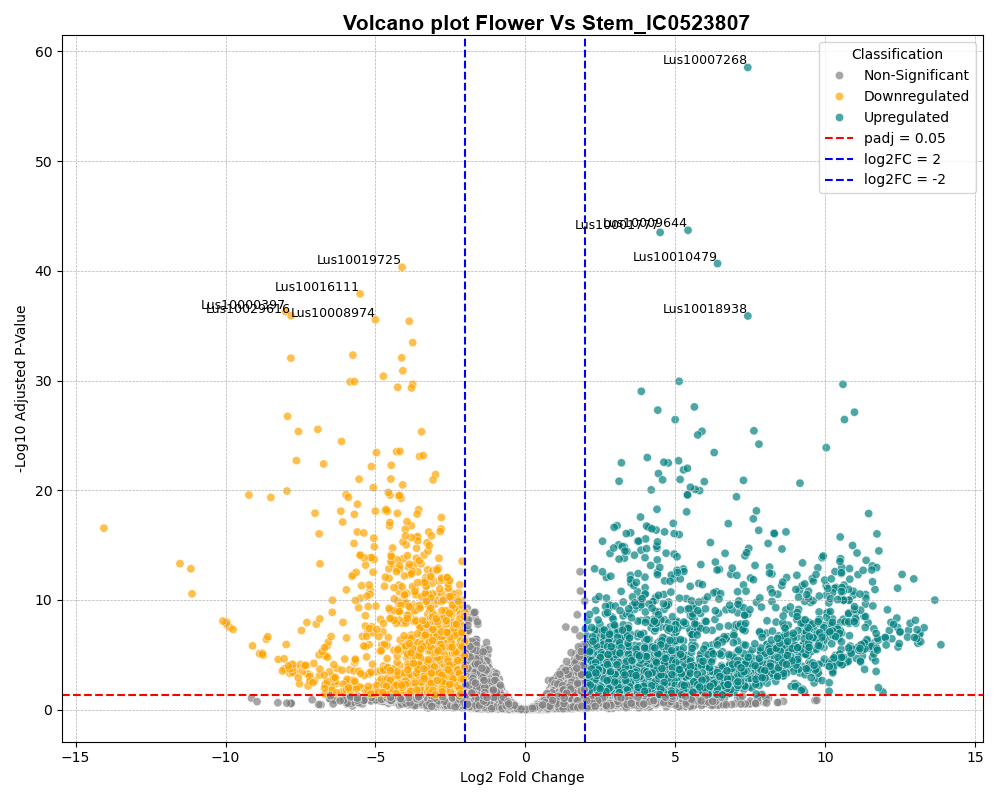
**

**Supplementary Figure S2g: Volcano plot depicting DEGs in comparison ‘Bud1 *vs* Leaf’ in accession IC0525939**

**
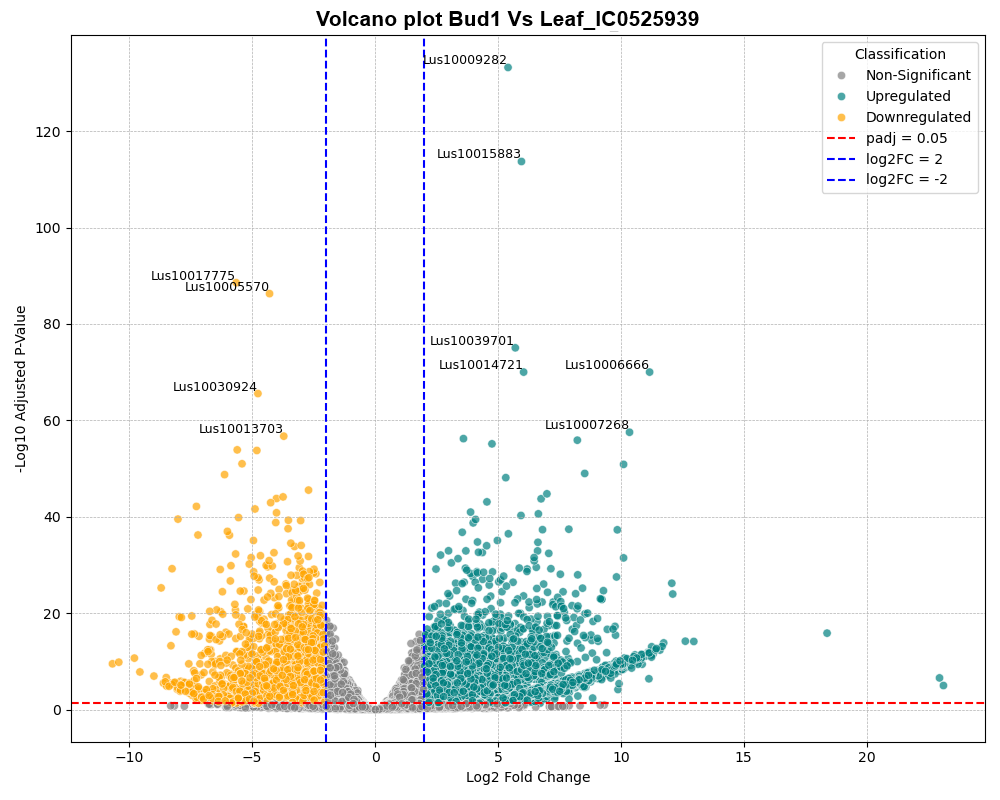
**

**Supplementary Figure S2h: Volcano plot depicting DEGs in comparison ‘Bud2 *vs* Leaf’ in accession IC0525939
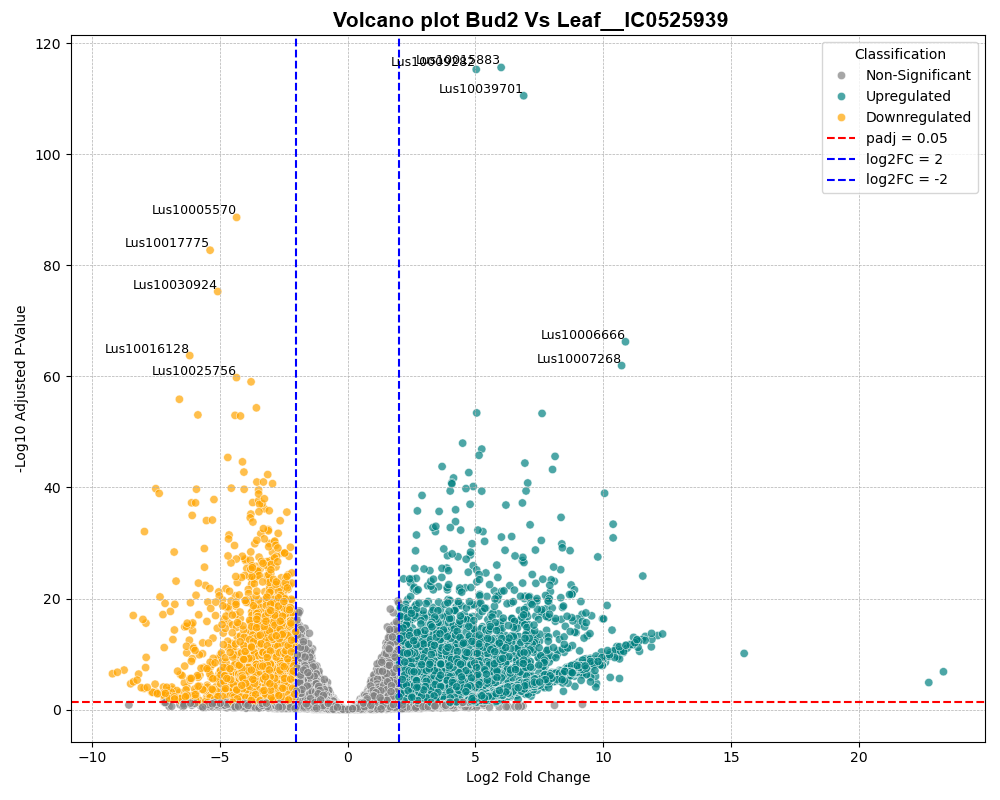
**

**Supplementary Figure S2i: Volcano plot depicting DEGs in comparison ‘Flower *vs* Leaf’ in accession IC0525939**

**
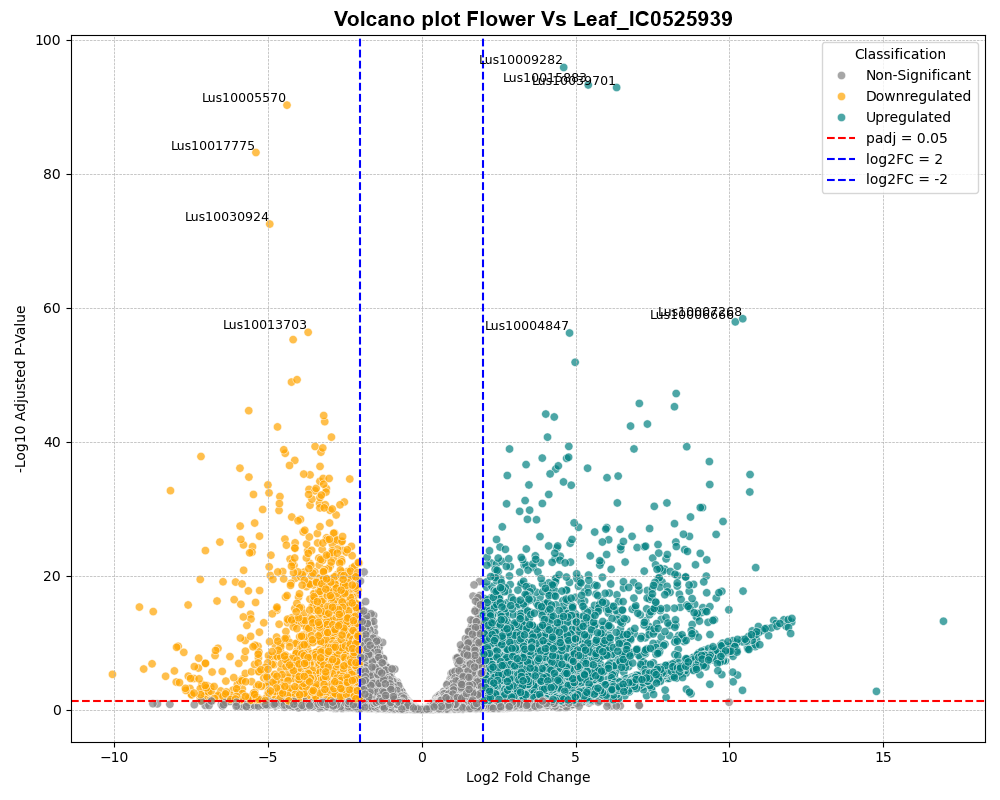
**

**
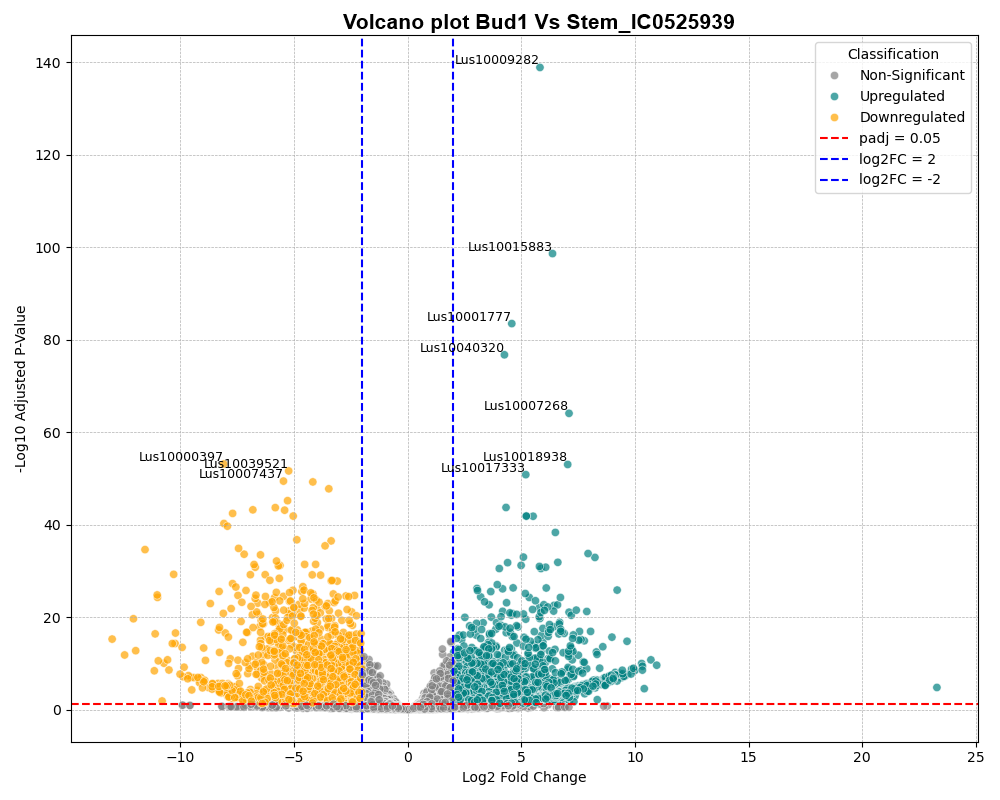
Supplementary Figure S2j: Volcano plot depicting DEGs in comparison ‘Bud1 *vs* Stem’ in accession IC0525939**

**Fig.S2k: Volcano plot depicting DEGs in comparison ‘Bud2 *vs* Stem’ in accession IC0525939**

**
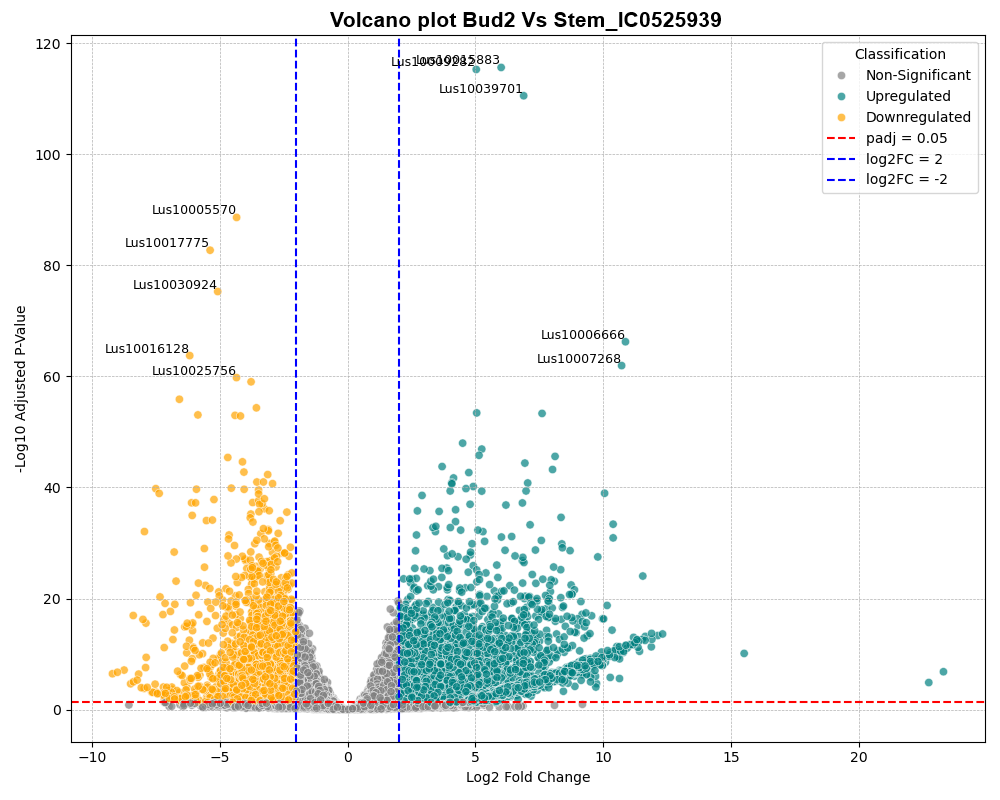
**

**Fig.S2l: Volcano plot depicting DEGs in comparison ‘Flower *vs* Stem’ in accession IC0525939**

**
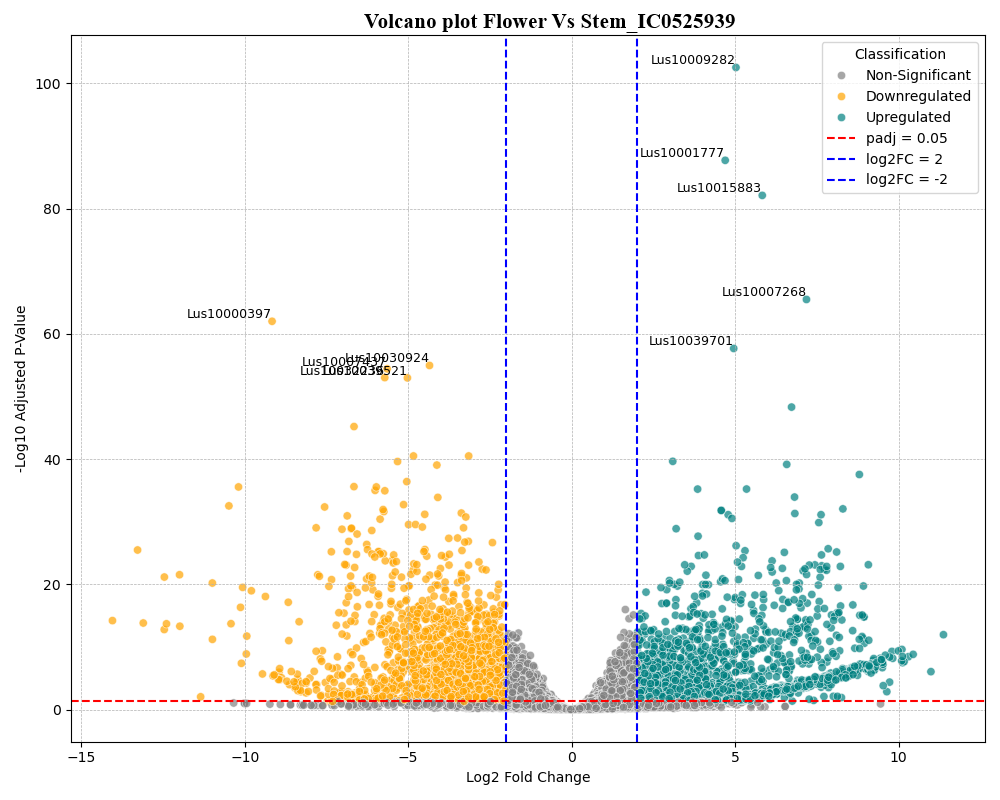
**

**Figure S3a: KEGG analyses of differentially expressed genes in plant hormone signal transduction pathway.**

**
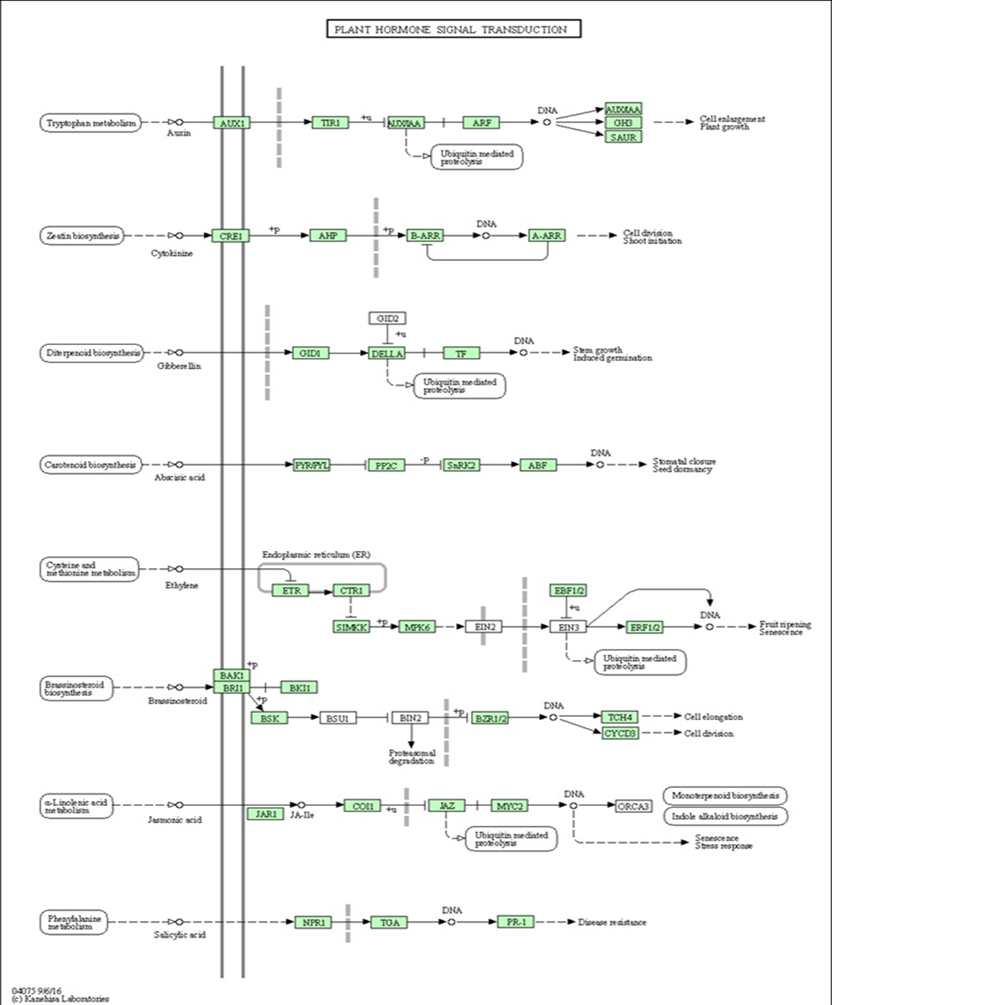
**

**Fig. S3b: KEGG analyses of differentially expressed genes in starch and sucrose metabolic pathway.**

**
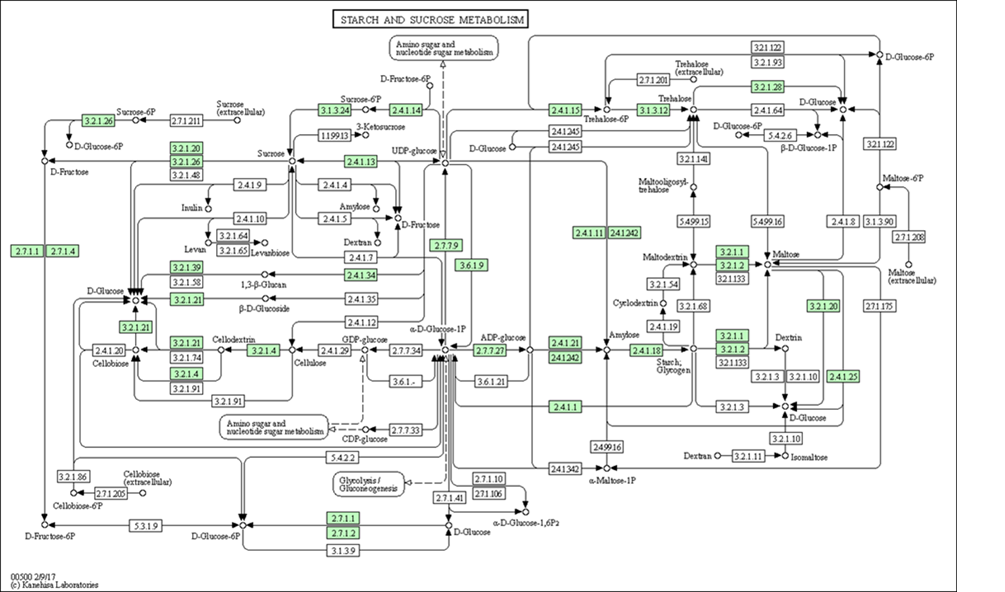
**

**Fig. S4: Top 20 enriched KEGG pathway terms of differentially expressed genes between reproductive tissues and vegetative tissues**

**
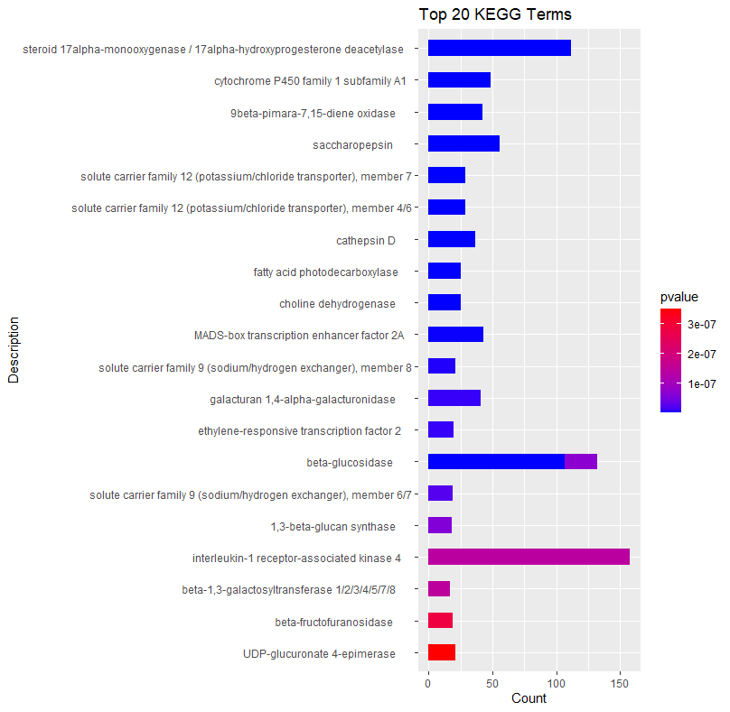
**

**Fig. S5 MapMan analyses of differentially expressed genes in hormone regulation, and redox metabolism.** Gene expression profile of DEGs in respective pathways are shown, blue color and red color represent upregulated and downregulated genes, respectively. The figure highlights differentially expressed genes from hormonal pathways, predominantly abscisic acid (ABA) and gibberellic acid (GA), along with redox-related pathways, in reproductive and floral tissues of the early flowering accessions.


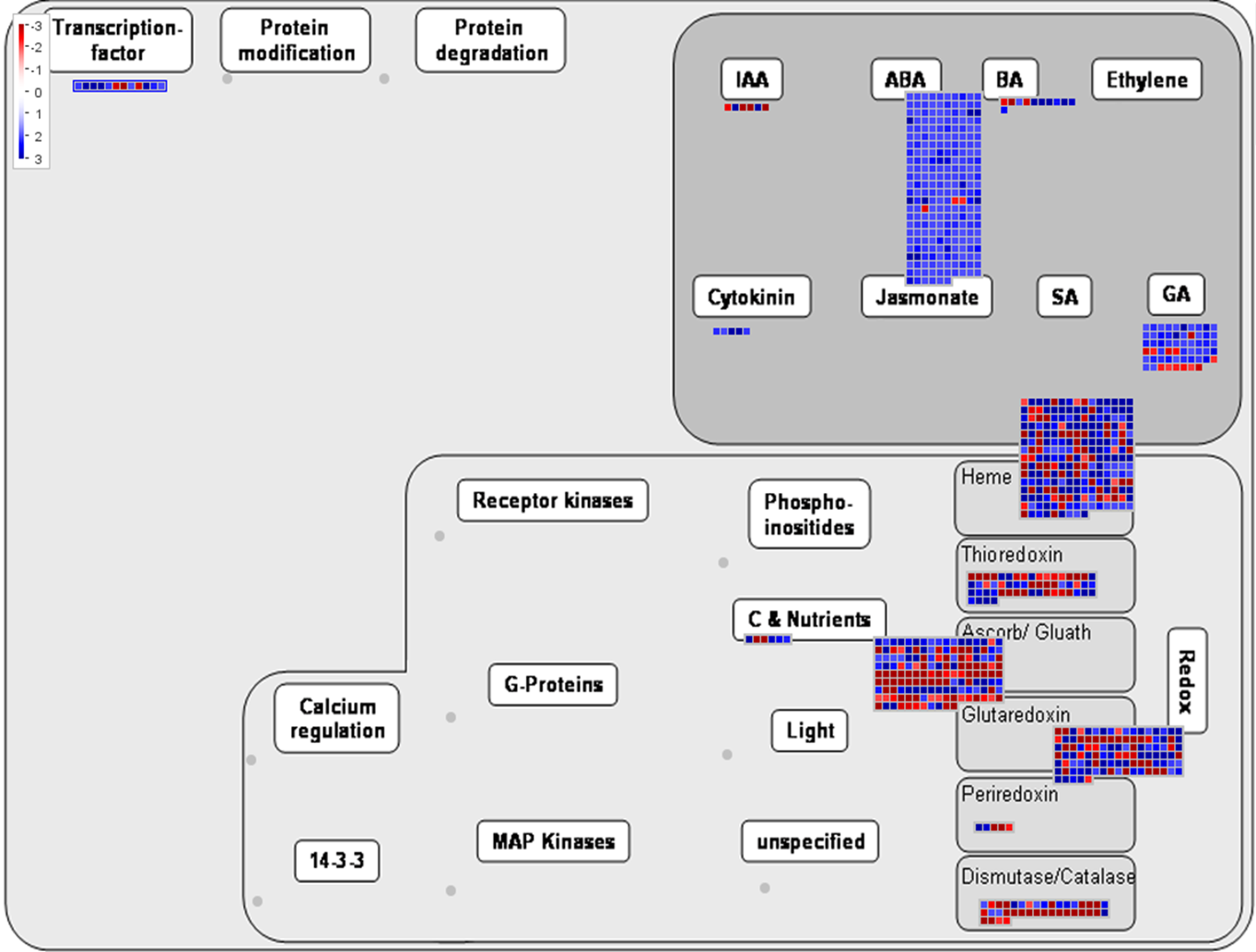


**Fig. S6: KEGG pathway analyses of differentially expressed genes. Under plant hormone signal transduction and circadian rhythm**

**
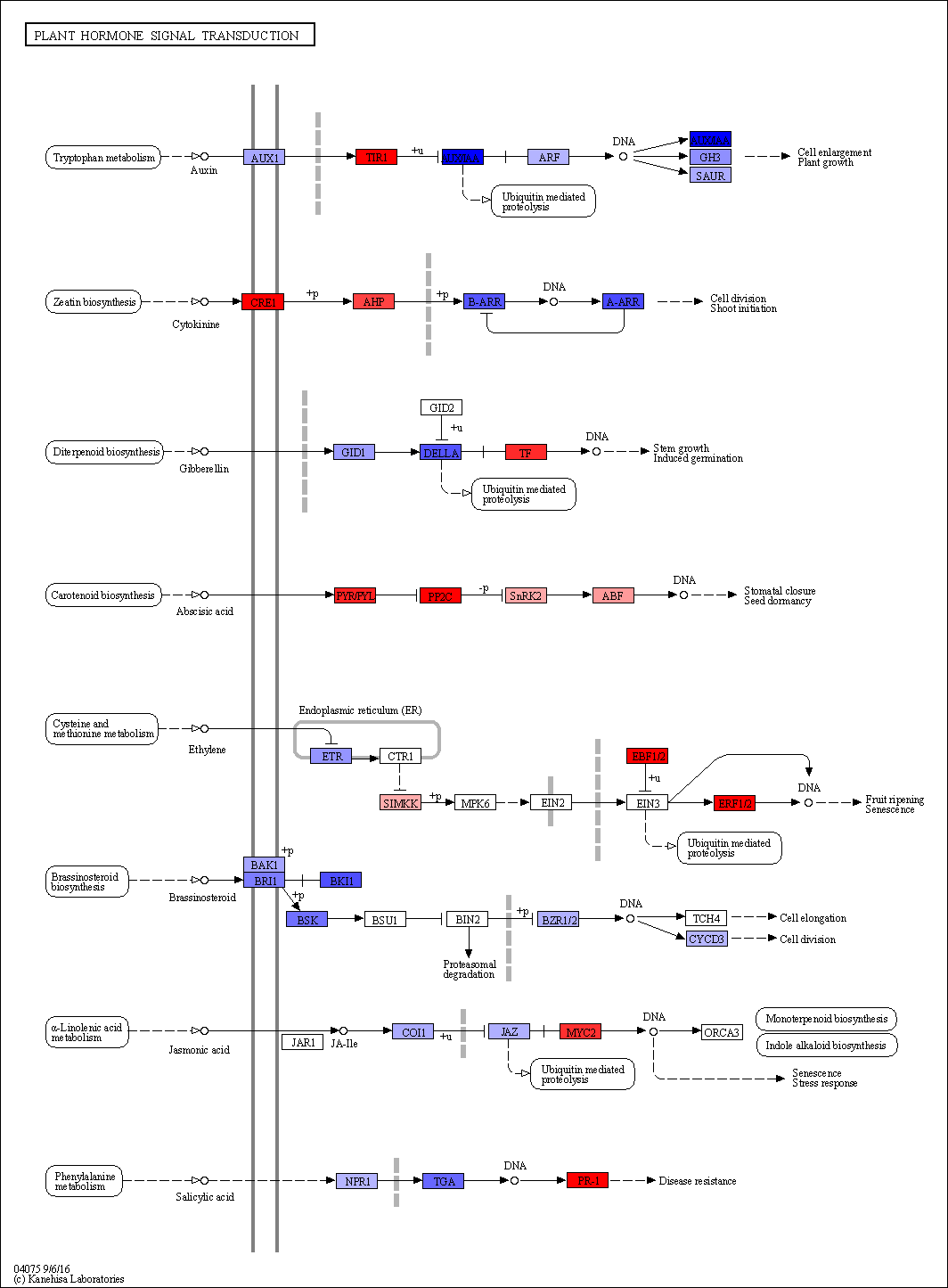
**

**Fig. S7. Pairwise correlation of gene expression between two biological replicates for the top 100 DEGs, flowering gene orthologs, and differentially expressed GWAS candidate genes.**

**
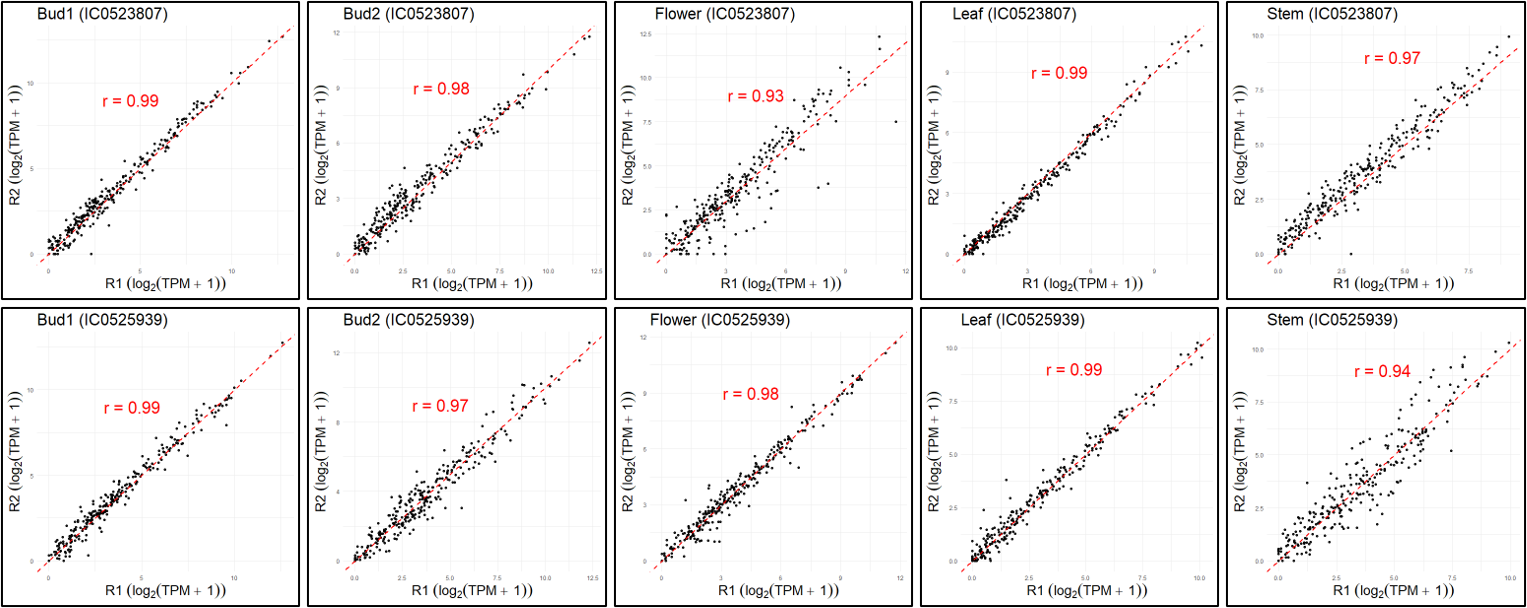
**
